# Lactate metabolism is essential in early-onset mitochondrial myopathy

**DOI:** 10.1126/sciadv.add3216

**Published:** 2023-01-04

**Authors:** Zhenkang Chen, Bogdan Bordieanu, Rushendhiran Kesavan, Nicholas P. Lesner, Siva Sai Krishna Venigalla, Spencer D. Shelton, Ralph J. DeBerardinis, Prashant Mishra

**Affiliations:** ^1^ Children’s Medical Center Research Institute, University of Texas Southwestern Medical Center, Dallas, TX 75390, USA.; ^2^ Abramson Family Cancer Research Institute, Perelman School of Medicine, University of Pennsylvania, Philadelphia, PA 19104, USA.; ^3^ Harold C. Simmons Comprehensive Cancer Center, University of Texas Southwestern Medical Center, Dallas, TX 75390, USA.; ^4^ Howard Hughes Medical Institute, University of Texas Southwestern Medical Center, Dallas, TX 75390, USA.; ^5^ Department of Pediatrics, University of Texas Southwestern Medical Center, Dallas, TX 75390, USA.

## Abstract

Myopathies secondary to mitochondrial electron transport chain (ETC) dysfunction can result in devastating disease. While the consequences of ETC defects have been extensively studied in culture, little in vivo data are available. Using a mouse model of severe, early-onset mitochondrial myopathy, we characterized the proteomic, transcriptomic, and metabolic characteristics of disease progression. Unexpectedly, ETC dysfunction in muscle results in reduced expression of glycolytic enzymes in our animal model and patient muscle biopsies. The decrease in glycolysis was mediated by loss of constitutive Hif1α signaling, down-regulation of the purine nucleotide cycle enzyme AMPD1, and activation of AMPK. In vivo isotope tracing experiments indicated that myopathic muscle relies on lactate import to supply central carbon metabolites. Inhibition of lactate import reduced steady-state levels of tricarboxylic acid cycle intermediates and compromised the life span of myopathic mice. These data indicate an unexpected mode of metabolic reprogramming in severe mitochondrial myopathy that regulates disease progression.

## Teaser

Metabolic analysis in an animal model of severe mitochondrial myopathy reveals a surprising dependence on lactate usage.

## INTRODUCTION

Genetic defects targeting the mitochondrial electron transport chain (ETC) are prevalent in the human population at an estimated incidence of greater than ~1:5000 and cause substantial morbidity and mortality (*1*, *2*). The clinical spectrum of mitochondrial disease is heterogeneous; however, most patients exhibit neuromuscular involvement, often with myopathic phenotypes as presenting symptoms (*3*, *4*). Although some nutrient supplements have shown promise in preclinical and initial clinical studies (*5*, *6*), there are currently no U.S. Food and Drug Administration (FDA)–approved therapies for mitochondrial myopathy (MM).

A major limitation to the study of MM is a paucity of informative animal models, which recapitulate the human phenotype. The “Deletor” mouse model, which makes use of a dominant *TWINKLE* mutation to initiate mitochondrial genome (mtDNA) deletions, has proved a useful model of adult, late-onset MM (*7*–*10*). Alternatively, conditional deletion of the complex IV assembly factors *Cox10* or *Cox15* in skeletal muscle results in a progressive myopathy and death at 6 to 9 months of age (*11*, *12*). Phenotypes in the commonly used *Ndufs4* knockout model of Leigh syndrome are driven by neuronal loss of complex I, and these mutant animals do not exhibit a muscular phenotype (*13*, *14*). Thus, there are limited animal models with severe, infantile forms of MM and few case-control human studies, resulting in the underlying disease physiology being largely unstudied. In vitro, severe mitochondrial ETC dysfunction can be modeled through multiple approaches, including the generation of mtDNA-deleted (rho0) cells, the presence of nuclear or mtDNA loss-of-function mutations, or drugs targeting the ETC. Almost universally, ETC dysfunctional cell lines up-regulate glycolytic flux, accompanied by increased secretion of lactate, and these cells are dependent on glucose for survival and maintenance of adenosine triphosphate (ATP) levels (*15*–*18*).

Here, we characterize the in vivo response to early-onset MM, making use of mice in which the mitochondrial dynamics genes *Mitofusin 1* (*Mfn1*) and *Mitofusin 2* (*Mfn2*) have been conditionally deleted in fast-twitch skeletal muscle (*19*). These mice exhibit biochemical, histological, phenotypic, and survival deficits characteristic of early-onset human MM and exhibit substantial metabolic alterations and energetic stress. Surprisingly and in contrast to in vitro models, we observed decreased expression and abundance of glycolytic enzymes and decreased glycolytic activity in affected muscle. A retrospective analysis of case-control studies in early-onset human MM revealed similar and highly correlative findings in which glycolytic enzymes were suppressed. Decreases in glycolytic enzymes are driven by loss of constitutive Hif1α signaling in affected muscle and result in extracellular lactate serving as a major carbon source. As a result, these severe MM mice are dependent on lactate import and utilization for survival. Our findings therefore indicate the mechanism underlying the loss of glycolytic enzymes in early-onset MM, which results in an unexpected nutrient dependency on lactate.

## RESULTS

### Glycolytic enzymes are reduced in a mouse model of severe MM

**Fig. 1.**
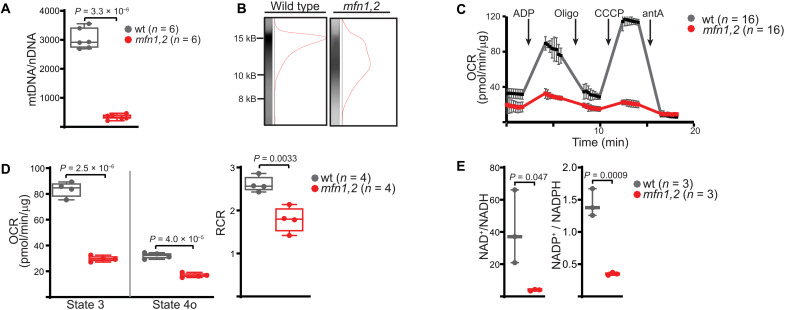
Deletion of *mfn1* and *mfn2* in fast-twitch skeletal muscle results in mitochondrial DNA depletion and respiratory defects. (**A**) Mitochondrial genome (mtDNA) content, normalized to nuclear genome content (nDNA) in TA muscle of the indicated genotype, measured by qPCR. (**B**) Representative long-range PCR results from the mitochondrial genome of TA muscle of the indicated genotype. In wt muscle, we observe a band of approximately 15 kilobases (kB). In *mfn1,2* muscle, we observe a smear of lower molecular weight (MW), consistent with amplification of multiple genomes. MW markers are indicated in kB. (**C**) Representative oxygen consumption rates (OCRs) from isolated mitochondria of TA muscles of the indicated genotype. The following compounds were injected at the indicated times: ADP, adenosine diphosphate; Oligo, oligomycin; CCCP, carbonyl cyanide 3-chlorophenylhydrazone; antA, antimycin A. Technical replicates from multiple wells are plotted. (**D**) Relative state 3 (ADP-stimulated) and state 4o (oligomycin inhibited) OCRs in isolated mitochondria from TA muscles of the indicated genotype. The respiratory control ratio (RCR) is indicated. (**E**) Redox metabolite ratios from TA muscles of wt and *mfn1,2* animals are presented. Box and whisker plots were plotted using the Tukey method. All data represent independent measurements from biological replicates, unless otherwise indicated. *P* values were calculated by two-tailed *t* test (A, D, and E), with adjustments for multiple comparisons.

**Fig. 2.**
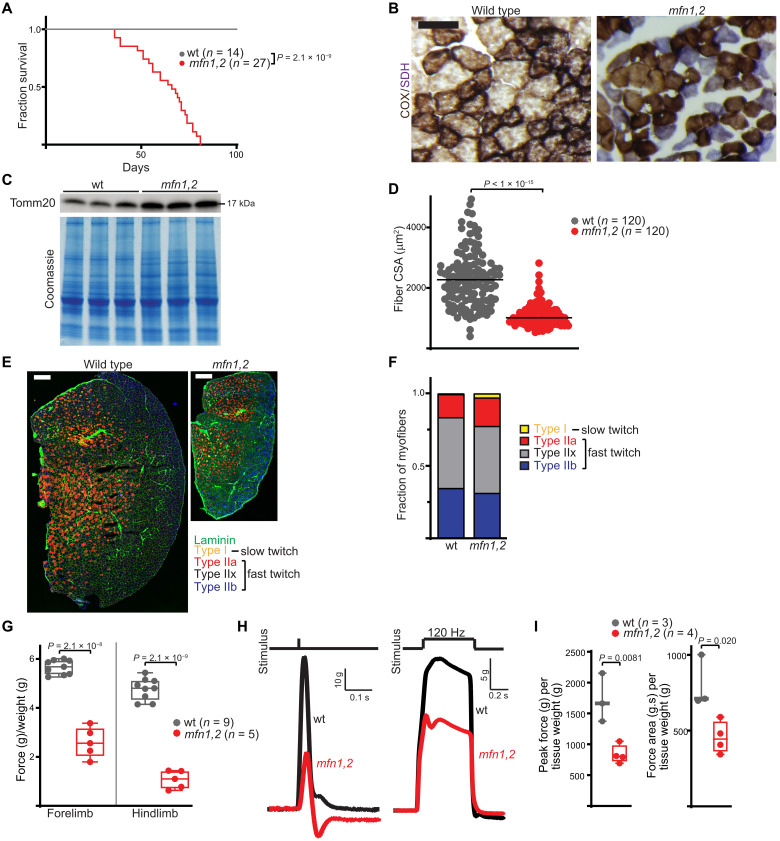
Deletion of *mfn1* and *mfn2* in fast-twitch skeletal muscle results in early-onset mitochondrial myopathy. (**A**) Fractional survival over age for animals of the indicated genotype. (**B**) Representative TA muscle cross sections from wt and *mfn1,2* animals, stained for COX activity (brown) and SDH activity (purple). Scale bar, 150 μm. (**C**) Western blot for Tomm20 (a mitochondrial outer membrane protein) from wt and *mfn1,2* TA muscle. A Coomassie-stained blot is shown as a loading control. MW markers are indicated in kDa. (**D**) Myofiber cross-sectional area (CSA) from TA muscles of the indicated genotype. (**E**) Representative immunofluorescent images of TA muscle cross-sections, stained for myofiber boundaries (laminin, green), and individual fiber types (types I, IIa, IIx, and IIb; colors indicated). Scale bars, 300 μm. (**F**) Quantitation of myofiber types as a fraction of total myofibers in wt and *mfn1,2* TA muscles. (**G**) Forelimb and hindlimb grip strength measured in wt and *mfn1,2* animals. (**H**) Representative traces of twitch (single stimulus) and tetanic (120 Hz stimulus) muscle force generated from in situ electrical stimulation of TA muscle. (**I**) Peak force and force area from tetanic stimulation of wt and *mfn1,2* muscle, normalized to tissue weight. Box and whisker plots were plotted using the Tukey method. All data represent independent measurements from biological replicates, unless otherwise indicated. *P* values were calculated by two-tailed *t* test (G and I), Mantel-Cox test (A), and Mann-Whitney test (D) with adjustments for multiple comparisons.

We characterized the in vivo response to early-onset MM, making use of mice in which the mitochondrial dynamics genes *Mfn1* and *Mfn2* have been conditionally deleted in fast-twitch skeletal muscle (*19*). Mitochondrial fusion is required for maintenance of the mitochondrial genome, and genetic co-deletion of *Mfn1* and *Mfn2* results in severe reduction in mtDNA quantity and quality in both cultured cells and muscle tissue (*19*, *20*). As previously reported (*19*), conditional deletion of *Mfn1* and *Mfn2* in fast-twitch muscle fibers using the Myl1-Cre driver (hereafter referred to as *mfn1,2*) (*21*) resulted in decreased mtDNA copy number and the appearance of mtDNA deletions detected by long-range polymerase chain reaction (PCR) (Fig. 1, A and B). Isolated mitochondria from the tibialis anterior (TA) muscle [composed of >98% fast-twitch muscle fibers (*22*)] exhibited significant reductions in oxygen consumption rates and the respiratory control ratio (Fig. 1, C and D). Correspondingly, TA muscle tissue from *mfn1,2* mice exhibited significant reductive stress, indicated by reductions in the NAD^+^ (nicotinamide adenine dinucleotide)/NADH (reduced form of NAD^+^) and NADP^+^ (nicotinamide adenine dinucleotide phosphate)/NADPH (reduced form of NADP^+^) ratios (Fig. 1E). Consistent with previous results, mutant mice had a shortened life span of less than 10 weeks (Fig. 2A), and affected muscles displayed histological defects including an increase in COX^−^SDH^+^ (cytochrome c oxidase negative, succinate dehydrogenase positive) myofibers, decreased myofiber size, and evidence of mitochondrial proliferation (Fig. 2, B to D). There were no notable changes in myofiber type (Fig. 2, E and F). Motor function analysis revealed significant deficits in grip strength, as well as ex vivo force generation (Fig. 2, G to I). Thus, *mfn1,2* animals exhibit biochemical, histological, functional, and survival deficits consistent with a severe early-onset MM.

**Fig. 3.**
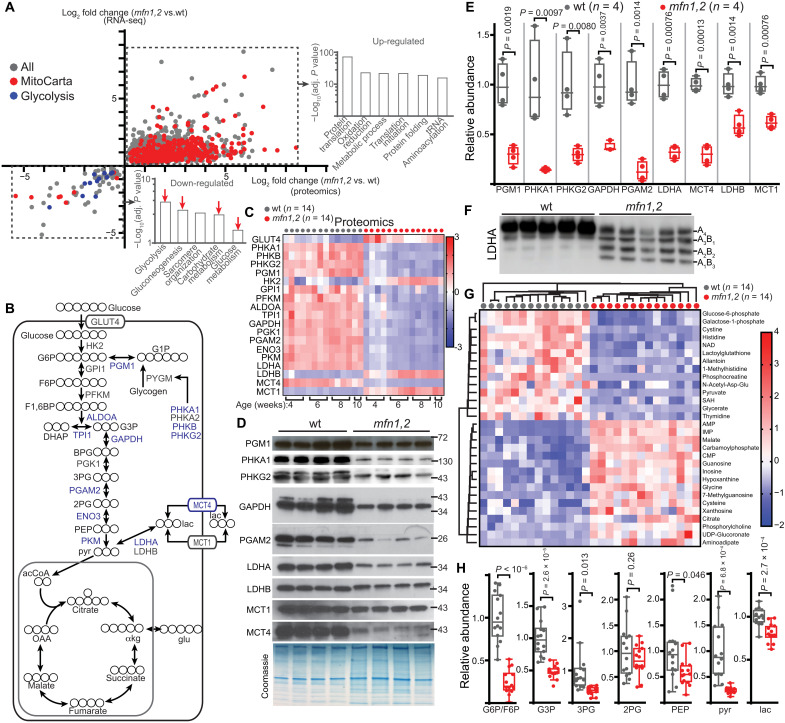
Decreased glycolytic enzymes in *mfn1,2* muscle. (**A**) Correlation of transcript and protein log_2_(fold changes) in *mfn1,2* versus wt muscle. Genes with significant transcript and protein changes in the same direction and the top enriched gene ontology pathways are shown. Glucose metabolic pathways (red arrows) are indicated. *n* = 14 animals per genotype. (**B**) Schematic of glucose and lactate metabolism in muscle. Metabolites are indicated with black circles (representing carbons). Glycolytic enzymes are colored blue (down-regulated in *mfn1,2* muscle) or gray (not down-regulated or not detected). (**C**) Protein abundances for muscle glycolytic genes, presented as a heatmap of *z* scores. (**D**) Western blot analysis of the indicated proteins. A Coomassie-stained gel is used as loading control. MW markers are indicated in kDa. (**E**) Quantitation of protein abundance, based on (D). (**F**) Native gel analysis of LDH tetramer from TA muscle visualized with an αLDHA antibody. The position of each tetramer is indicated. (**G**) Heatmap (*z*-score values) for the top 30 changing metabolites between wt and *mfn1,2* muscle. (**H**) Relative abundances of the indicated glycolytic metabolites in wt and *mfn1,2* muscle. Same legend as (G). Box and whisker plots are plotted using the Tukey method. All data represent independent measurements from biological replicates. *P* values were calculated by two-tailed *t* test (E and H), with adjustments for multiple comparisons. G1P, glucose-1-phosphate; G6P, glucose-6-phosphate; F6P, fructose-6-phosphate; F1,6BP, fructose-1,6-biphosphate; DHAP, dihydroxyacetone phosphate; G3P, glyceraldehyde-3-phosphate; BPG, 2,3-biphosphoglycerate; 3PG, 3-phosphoglycerate; 2PG, 2-phosphoglycerate; PEP, phosphoenolpyruvate; pyr, pyruvate; lac, lactate; acCoA, acetyl-CoA; OAA, oxaloacetate; cit, citrate; αkg, α-ketoglutarate; glu, glutamate; fum, fumarate; mal, malate.

To understand the in vivo adaptations in the *mfn1,2* mouse model, we first profiled TA muscle from age-matched wild-type (wt) and affected littermates, using a combination of transcriptomics and proteomics. Unsupervised hierarchical clustering of normalized RNA-sequencing (RNA-seq) values separated wt and *mfn1,2* samples (fig. S1A), and differential expression analysis revealed a large number of altered genes (adjusted *P* < 0.01; |log_2_(fold change)| > 1), including 849 up-regulated and 877 down-regulated genes (fig. S1B and table S1). Similarly, unsupervised hierarchical clustering distinguished the proteomes of wt and *mfn1,2* muscle (fig. S1C); here, there was a substantial enrichment of up-regulated (1340) proteins, as compared with down-regulated (123) proteins (fig. S1D and table S1). Thus, *mfn1,2* muscle exhibits substantial transcriptome and proteome alterations from wt muscle. We focused on high-confidence (adjusted *P* < 0.01) genes that were similarly regulated (|log_2_(fold change)| > 1) in both our RNA-seq and proteomic datasets (Fig. 3A). Consistent with the accumulation of mitochondria in *mfn1,2* muscles (Fig. 2, B and C), gene ontology analysis of up-regulated genes indicated enrichment for biological processes related to translation and oxidation reduction, including a notable enrichment of mitochondrial genes (based on MitoCarta 3.0 classification) (Fig. 3A). We noted that mtDNA-encoded genes exhibited depletion as early as 4 weeks of age (fig. S1E), consistent with the deficits in mitochondrial genome content in *mfn1,2* muscle (Fig. 1, A and B). In addition, the integrated mitochondrial stress response gene set was highly up-regulated in *mfn1,2* muscle at all ages, similar to adult-onset models of MM (fig. S1F) (*8*).

Gene ontology analysis of down-regulated genes indicated enrichment of biological processes involved in glycolysis or glucose metabolism (Fig. 3A, red arrows). The down-regulation of genes and proteins in the glycolysis pathway was surprising considering the near-universal finding of enhanced glycolytic flux in the setting of various types of mitochondrial dysfunction in vitro. We therefore assessed abundances of the major muscle glycolytic enzymes in our datasets and found that the majority (15 of 19 detected proteins) showed evidence of depletion as early as 4 weeks of age (Fig. 3, B, blue labels, and C). We note that four glycolytic enzymes (GLUT4, HK2, LDHB, and MCT1) were not depleted (Fig. 3C). We validated a subset of these findings by Western blot analysis, which indicated significant depletion of glycogenolytic enzymes (PGM1, PHKA1, and PHKG2), the upper glycolytic enzymes (GAPDH and PGAM2), and the lactate handling enzymes (LDHA and MCT4) (Fig. 3, D and E). In contrast, LDHB and MCT1 were less affected in *mfn1,2* muscle (Fig. 3, D and E). Given the changes in LDHA levels, we investigated the composition of the lactate dehydrogenase enzyme. Lactate dehydrogenase functions as a tetramer, composed of LDHA and LDHB in various ratios (*23*). In wt muscle, native gel analysis indicated that the vast majority of LDH is present as the LDHA_4_ tetramer (Fig. 3F). In *mfn1,2* muscle, the substantial decrease in LDHA results in a shift to LDHB-containing tetramers (Fig. 3F).

Although the reduced levels of some glycolytic enzymes were surprising, enzyme amounts are not directly indicative of metabolic activity. Some key glycolytic enzymes (e.g., GLUT4 and HK2) are not depleted but are instead up-regulated (Fig. 3C). While we primarily focused on the major muscle isoforms of these genes, it is possible that up-regulation in alternative isoforms is compensating for the decrease in the muscle isoforms. To examine this, we assessed changes in transcript and protein abundance in all detectable isoforms of each glycolytic enzyme (fig. S2A). We did observe that some alternative isoforms were up-regulated in *mfn1,2* muscle, including GLUT1, HK1, PFKP, PFKB2, PFKB4, PGAM1, PGAM5, and ENO1 (fig. S2A). However, the abundances of these alternative isoforms were usually several fold below the levels of the major muscle isoforms. In addition, the glycolysis rate-limiting enzyme phosphofructokinase muscle isoform (PFKM) was down-regulated, and the levels of PFK regulators (PFKB1 to PKFB4) were largely unchanged (fig. S2A). Thus, the abundance of muscle isoforms of glycolytic enzymes tends to be down-regulated, without large compensatory changes in the alternative isoforms.

To address metabolism more directly, we first profiled steady-state metabolite levels in plasma and TA muscle from wt and *mfn1,2* littermates (table S2). Principal components analysis did not reveal distinctions between the plasma metabolomes (fig. S3A), and we did not detect elevated lactate or pyruvate in *mfn1,2* plasma (fig. S3B). In contrast, the metabolome of *mfn1,2* muscle was altered from that of wt muscle, as indicated by principal components analysis (fig. S3A). Of the 187 detected metabolites, steady-state levels of a large proportion (64 metabolites, 34%) were significantly altered (table S2). A number of purine-related metabolites were present among the up-regulated metabolites (Fig. 3G), consistent with altered one-carbon metabolism and deoxynucleoside triphosphate (dNTP) pools, also observed in animal models of adult-onset MM (*9*). In contrast, several glycolytic intermediates were reduced in *mfn1,2* muscle (Fig. 3, G and H), and we did not detect elevation of lactate in *mfn1,2* muscle. We noted that mitochondrial tricarboxylic acid (TCA) cycle metabolites were largely preserved or increased in affected muscle (fig. S3C). Thus, steady-state analysis of *mfn1,2* muscle reveals depletion of both muscle glycolytic enzymes and metabolites, concomitant with elevation of mitochondrial proteins and sparing of mitochondrial TCA cycle metabolites.

**Fig. 4.**
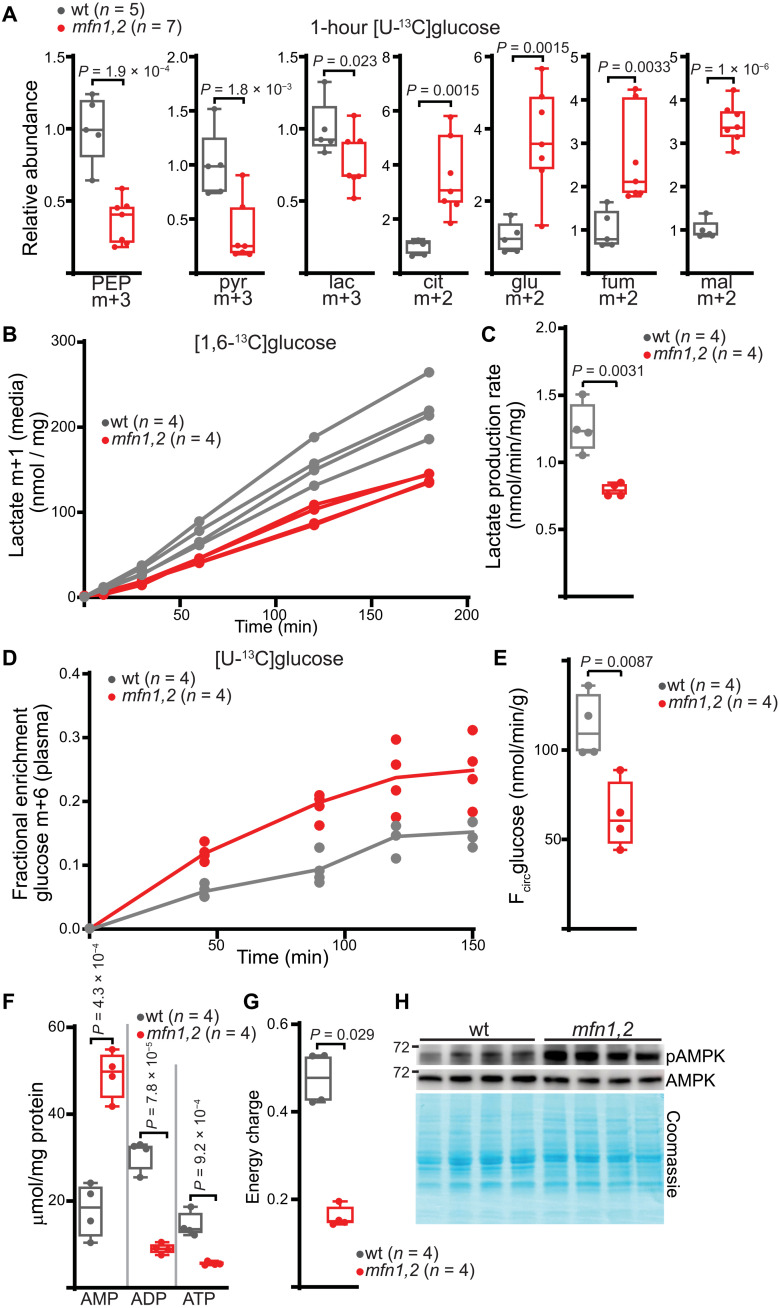
Reduced glycolysis in *mfn1,2* muscle. (**A**) Relative isotopolog abundance of the indicated labeled species following a 1-hour [U-^13^C]glucose challenge. (**B**) Lactate production versus time in ex vivo isolated muscle preparations incubated with [1,6-^13^C]glucose, normalized to TA muscle weight. (**C**) Lactate production rates from ex vivo isolated muscle preparations. (**D**) Fractional enrichment in plasma glucose m+6 from animals infused with [U-^13^C]glucose. (**E**) Glucose whole-body turnover rates (F_circ_glucose) from animals of the indicated genotype. (**F**) Absolute levels of AMP, ADP, and ATP in wt and *mfn1,2* muscle, normalized to total protein amount. (**G**) Energy charge ([ATP] + 1/2[ADP])/([ATP] + [ADP] + [AMP]) in wt and *mfn1,2* muscle. (**H**) Representative Western blot for AMPK and pAMPK in indicated muscle samples. Coomassie-stained proteins are shown as a loading control. MW markers are indicated in kDa. In all panels, wt data are represented in gray, and *mfn1,2* data are represented in red. Box and whisker plots are plotted using the Tukey method. All data represent independent measurements from biological replicates. *P* values were calculated by multiple two-tailed *t* test (A and F) or two-tailed *t* test (C, E, and G), with adjustments for multiple comparisons. PEP, phosphoenolypyruvate; pyr, pyruvate; lac, lactate; cit, citrate; glu, glutamate; fum, fumarate; mal, malate.

Methods to directly measure glycolytic flux in vivo are not yet developed. To probe the relative glycolytic activity between wt and *mfn1,2* animals, we performed a number of independent analyses. First, we investigated turnover of glycolytic intermediates by subjecting animals to a single dose of universally ^13^C labeled glucose ([U-^13^C]glucose) at 4 weeks of age (before the onset of survival deficits), followed by measurement of labeled glycolytic intermediates 1 hour later (Fig. 4A). The presence of heavy m+3 species of glycolytic metabolites indicates utilization of the labeled glucose, while m+2-labeled species in the TCA cycle indicate contribution of pyruvate to mitochondrial metabolites via pyruvate dehydrogenase. Quantitation of m+3 PEP (phosphoenolpyruvate), pyruvate, and lactate species revealed significantly decreased isotopolog abundances in *mfn1,2* muscle (Fig. 4A). These decreased abundances were not due to increased turnover of labeled metabolites, as we did not observe increased fractional enrichment in PEP or pyruvate species (fig. S3E). In contrast, the abundance of labeled mitochondrial metabolites was significantly increased in affected tissue (Fig. 4A). Together, these results suggest reduced glucose contribution to glycolytic intermediates in *mfn1,2* muscle; however, glucose is a more prominent carbon source for mitochondrial metabolites under these conditions (discussed more below).

A precise interpretation of a single bolus of [U-^13^C]glucose is complicated by a myriad of parameters, including differences in circulating metabolites (fig. S3D), individual enrichments (fig. S3E), and pool size (fig. S3F). We therefore performed experiments to better assess glucose utilization in wt and *mfn1,2* muscle. First, we acutely dissected intact TA muscles from 4-week-old animals and immediately incubated them in glucose-free medium supplemented with 10 mM [1,6-^13^C]glucose. By following lactate m+1 production in the medium (Fig. 4B) over time, we could directly quantitate glycolytic flux. *mfn1,2* muscles exhibited a significant reduction in lactate production rates (Fig. 4C), indicating that the constellation of proteomic changes in glycolytic enzymes is correlated with decreased glycolytic activity. To complement this ex vivo measurement, we performed steady-state infusions of [U-^13^C]glucose to calculate the in vivo whole-body turnover flux of glucose (F_circ_glucose) (Fig. 4D) (*24*). We observed that *mfn1,2* animals have a significant decrease in F_circ_glucose (Fig. 4E), suggesting that changes in glycolytic activity of fast-twitch skeletal muscle can influence the whole-body turnover rate. However, this experiment does not rule out the possibility that other tissues have adjusted their glucose utilization in response to the genetic perturbation. Together, these experiments are not consistent with elevated glycolysis in the setting of early-onset myopathy and instead suggest that glycolytic rates are suppressed in this disease model.

As glucose utilization by skeletal muscle can be regulated by insulin, we investigated insulin and glucagon levels in plasma of wt and *mfn1,2* animals (fig. S3G), which did not reveal significant differences. In addition, we did not observe deficits in a glucose tolerance test or insulin tolerance test in *mfn1,2* animals (fig. S3, H and I). Reduced glycolysis, combined with a mitochondrial respiratory defect in these animals (Fig. 1C), is expected to result in an energy deficit. Absolute values of adenosine diphosphate (ADP) and ATP were significantly reduced in *mfn1,2* muscle, concomitant with a significant increase in adenosine monophosphate (AMP) levels (Fig. 4F). Correspondingly, we observed a significant reduction in the energy charge, as well as increased phosphorylation of AMP kinase (AMPK) in *mfn1,2* muscles (Fig. 4, G and H). As the metabolism of fast-twitch muscle can be activity dependent, we repeated these analyses after an exercise challenge (fig. S4). Animals were first subjected to 30 minutes of moderate treadmill exercise; muscle was then immediately dissected and frozen after exercise. We observed similar elevations of pAMPK (phosphorylated AMPK) and a reduction of energy charge in *mfn1,2* animals (fig. S4, A and B). The steady-state post-exercise metabolome was altered in *mfn1,2* animals (fig. S4C), and a detailed analysis revealed similar depletions of a number of glycolytic intermediates, as well as elevation of TCA cycle intermediates (fig. S4D). Thus, affected muscle in this early-onset myopathic model appears similarly affected in sedentary and exercised states.

### Glycolytic enzymes are reduced in early-onset human MM

**Fig. 5.**
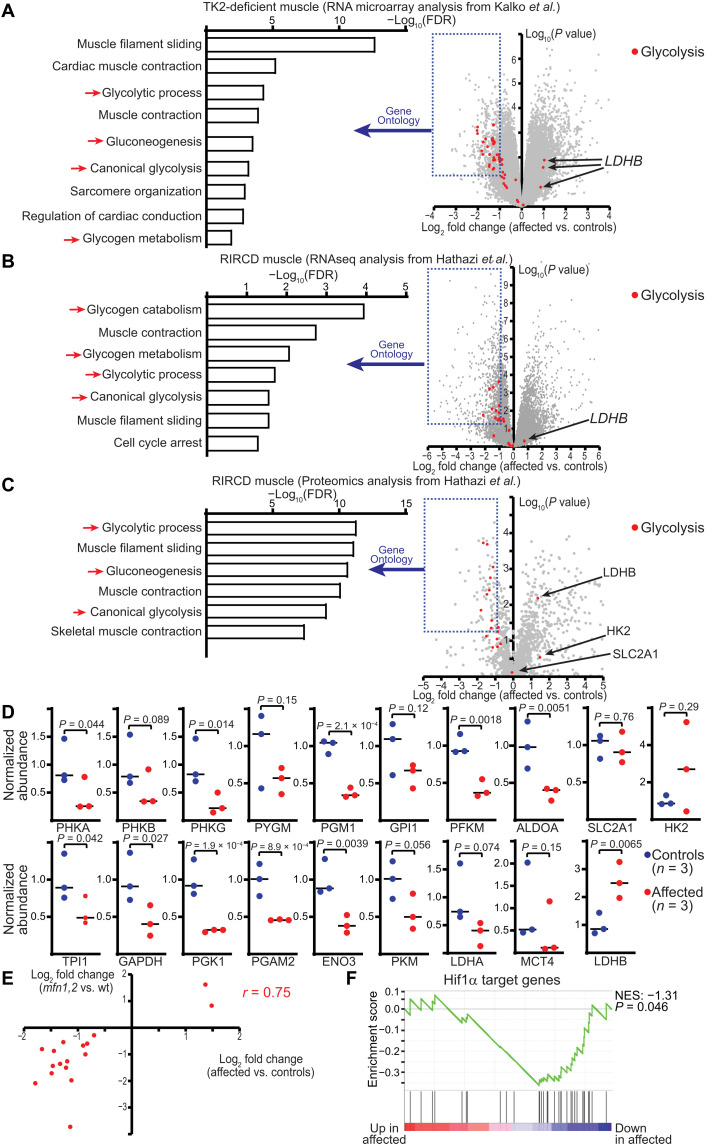
Transcriptomic and proteomic analysis of muscle biopsies from case-control studies in early-onset mitochondrial myopathy. (**A**) Volcano plot of probe intensities from RNA microarray analysis in affected versus control muscle biopsies from patients with TK2-deficient MM, assessed from data collected by Kalko *et al*. (*25*), and available at NCBI GEO GSE43698. Log_2_(fold change) is plotted against the −log_10_(adjusted *P* value) for each gene probe. Probes against muscle glycolytic enzymes are indicated in red. Gene ontology analysis is shown for down-regulated genes (blue box), and the significant (FDR < 0.05) biological processes are indicated. Red arrows indicate glucose and glycogen metabolic pathways. (**B**) Same as (A), except for RNA-seq data obtained from affected versus control muscle biopsies in patients with RIRCD, collected by Hathazi *et al*. (*26*). (**C**) Same as (A), except for proteomic abundance data from affected versus control muscle biopsies in patients with RIRCD, collected by Hathazi *et al*. (**D**) Normalized protein abundances of the indicated glycolytic enzymes based on proteomics data in RIRCD muscle biopsies. (**E**) Correlation between fold changes in the proteomic abundance of glycolytic enzymes in affected muscle of the *mfn1,2* animal model (*y* axis) versus RIRCD muscles (*x* axis). The Pearson correlation coefficient is indicated. (**F**) GSEA of Hif1α target genes in affected versus control RIRCD muscle biopsies. NES, normalized enrichment score, and *P* value are indicated.

As it was surprising to find down-regulation of glycolytic enzymes at the protein and mRNA levels, we investigated the extent to which this phenomenon occurs in human patient samples (table S3). Severe, infantile MMs are rare entities typically due to mutations in genes affecting the mitochondrial ETC. An exhaustive search of the literature identified two independent case-control studies in which muscle biopsy samples had been obtained from affected patients and age-matched controls. The first study (*25*) investigated muscle transcriptomes in four patients with 90% mtDNA depletion due to mutations in TK2 (thymidine kinase 2). We reassessed their gene microarray data (Fig. 5A) and performed gene ontology analysis on significantly down-regulated genes [log_2_(fold change) < −1; *P* < 0.05]. Similar to data from our animal model, we observed enrichment of several biological processes related to glucose and glycogen metabolism (Fig. 5A, red arrows). Most of the muscle glycolytic enzymes were down-regulated, and the only transcript with evidence of up-regulation was LDHB (Fig. 5A, red dots).

A second study (*26*) focused on a cohort of pediatric patients suffering from RIRCD (reversible infantile respiratory chain deficiency), a rare disease due to an mtDNA mutation that presents with severe infantile myopathy. In this study, the authors performed RNA-seq and proteomics analysis on muscle biopsies from affected patients and controls. We reassessed their datasets and similarly performed gene ontology analysis on down-regulated transcripts or proteins (Fig. 5, B and C). Again, we observed enrichment of biological processes related to glucose and glycogen metabolism (Fig. 5, B and C, red arrows), and glycolytic enzymes were largely down-regulated at both the transcript and protein level (Fig. 5, B and C, red dots). Normalized proteome levels of glycolytic enzymes revealed lower abundance in patient samples (Fig. 5D), with the exception of HK2, LDHB, and SLC2A1. As described above, these enzymes were also not down-regulated in our animal model (Fig. 3, C to E). The fold change in protein abundance observed for glycolytic enzymes in our animal model was highly correlated with the fold change observed in patient samples (Fig. 5E). Thus, in these two unrelated datasets from patients affected by early-onset MM, we observe a very similar down-regulation of most glycolytic pathway enzymes as observed in our *mfn1,2* model, with the same orthologous proteins (e.g., SLC2A1, HK2, and LDHB) as exceptions to this trend.

### Glucose handling is perturbed in *mfn1,2* muscle

**Fig. 6.**
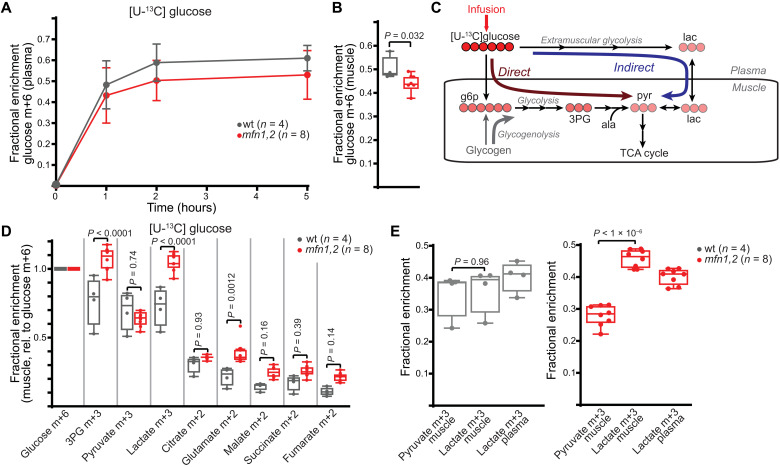
Altered glucose contribution to central carbon metabolism in *mfn1,2* muscle. (**A**) Plasma labeling of glucose m+6 in steady-state infusion experiments with [U-^13^C]glucose. (**B**) Glucose m+6 enrichment in muscle from steady-state [U-^13^C]glucose infusions. Same legend as (A). (**C**) Overview of direct and indirect pathways for glucose contribution to muscle metabolites. The infused compound is labeled with a red arrow, and filled red circles refer to heavy carbons (^13^C). Dilution of labeling due to glycogenolysis is indicated with the gray arrow. (**D**) Fractional enrichment of the indicated labeled species in TA muscle from steady-state [U-^13^C]glucose infusions. Data are normalized to muscle glucose m+6 enrichment values. (**E**) Fractional enrichment in the indicated muscle and plasma species from steady-state [U-^13^C]glucose infusions. In all panels, wt data are represented in gray, and *mfn1,2* data are represented in red. Box and whisker plots were plotted using the Tukey method. All data represent independent measurements from biological replicates. *P* values were calculated by multiple two-tailed *t* test (D), one-way ANOVA (E), or two-tailed *t* test (B), with adjustments for multiple comparisons. g6p, glucose-6-phosphate; ala, alanine; pyr, pyruvate; lac, lactate.

To investigate central carbon utilization in wt versus *mfn1,2* muscle, we made use of steady-state isotopic infusion techniques in conscious animals (*27*, *28*). Steady-state euglycemic infusion of [U-^13^C]glucose (Fig. 6, A to C) was used to assess glucose contribution to glycolytic and TCA cycle metabolites. We observed elevated labeling in 3-phosphoglycerate (3PG), a late glycolytic intermediate in *mfn1,2* muscles (Fig. 6D). In this experiment, the 3PG enrichment is typically decreased (relative to glucose enrichment) due to dilution from the breakdown of unlabeled glycogen (Fig. 6C, gray arrow) (*29*). The elevated 3PG labeling observed in *mfn1,2* animals is consistent with an impairment in glycogenolysis, possibly due to the down-regulation of glycogenolytic enzymes observed in affected muscle (Fig. 3, C to E). There were no significant differences between glycogen levels of wt and *mfn1,2* muscle (fig. S5A).

Labeled glucose potentially contributes to muscle metabolites through two pathways (Fig. 6C). First, “direct” import of glucose into muscle, followed by glycolysis, can lead to glucose-derived carbons contributing to pyruvate, lactate, and TCA cycle metabolites. Alternatively, extramuscular glycolysis can result in labeled lactate in the plasma, which can be imported into muscles and oxidized (referred to as the “indirect” pathway) (Fig. 6C). Differences in the enrichment of muscle metabolites during a ^13^C-glucose infusion might be reflective of alterations in the relative fluxes of these two pathways. We observed that the contribution of glucose-derived carbons to lactate was substantially increased over contribution to pyruvate in *mfn1,2* muscle, indicating that muscle pyruvate is not the major source of muscle lactate in this disease model, and these two metabolites are not in fast exchange (Fig. 6, D and E). Instead, muscle lactate more closely matched the enrichment in plasma lactate in *mfn1,2* animals (Fig. 6E). In addition, glucose contribution to TCA cycle metabolites was elevated in *mfn1,2* versus wt muscle, which is consistent with an elevated relative flux of lactate import and oxidation (Fig. 6D). Because glucose labeling can result in scrambling of labels, we also assessed total labeling of muscle metabolites, which yielded similar results (fig. S5B). These results are reminiscent of findings in some human tumors and mouse tissues in which other labeled carbon sources (e.g., lactate) are imported and contribute to TCA cycle metabolites (*24*, *30*, *31*).

### Lactate is metabolized in *mfn1,2* muscle

Because of the observed perturbations in glucose handling, we therefore investigated the potential contribution of other carbon sources to central carbon metabolism. Steady-state infusion of labeled alanine resulted in ~15% enrichment in plasma samples (fig. S5C); however, muscle labeling of alanine was substantially lower (~2% enrichment) in both wt and *mfn1,2* animals, indicating that import is not a major source of alanine in fast-twitch muscle (fig. S5D). Relative to muscle labeling of alanine, we noted no significant differences in contribution to pyruvate, lactate, or TCA cycle metabolites between wt and *mfn1,2* muscle (fig. S5E).

**Fig. 7.**
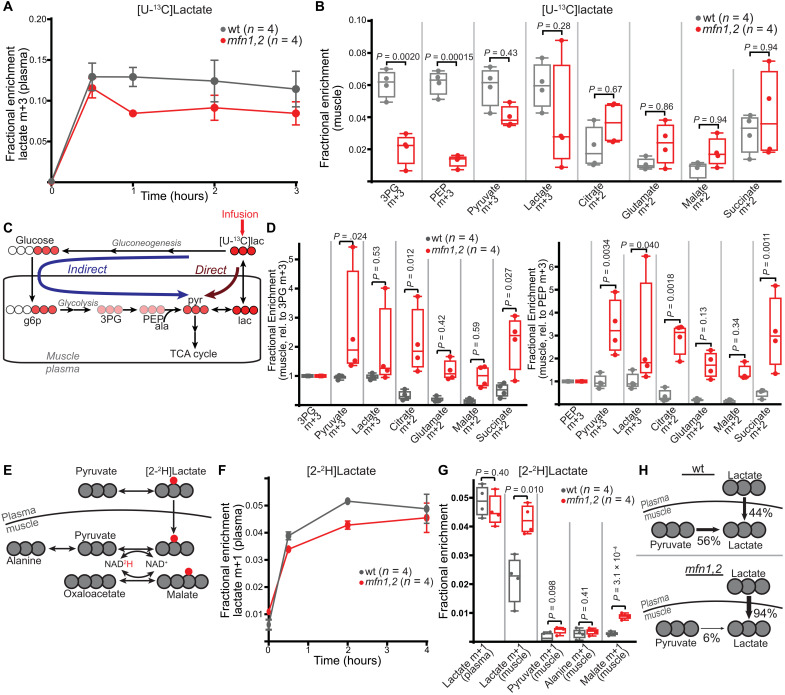
Enhanced contribution from lactate to central carbon metabolism in *mfn1,2* muscle. (**A**) Plasma labeling of lactate m+3 in steady-state infusion experiments with [U-^13^C]lactate. (**B**) Fractional enrichment of the indicated labeled species in TA muscle from steady-state [U-^13^C]lactate infusions. (**C**) Overview of direct and indirect pathways for lactate contribution to muscle metabolites. The infused compound is labeled with a red arrow, and filled red circles refer to heavy carbons (^13^C). (**D**) Fractional enrichment of the indicated labeled species in muscle from steady-state [U-^13^C]lactate infusion. Data are normalized to muscle 3PG m+3 (left) or PEP m+3 (right) enrichment values. (**E**) Schematic of expected labeling pattern from [2-^2^H]lactate infusion experiments. Carbon atoms are shown as gray circles; the labeled deuterium is shown in red. (**F**) Plasma labeling of lactate m+1 in steady-state infusion experiments with [2-^2^H]lactate. (**G**) Fractional enrichment of the indicated labeled species in TA muscle from steady-state [2-^2^H]lactate infusion. (**H**) Summary of relative contributions to muscle lactate from pyruvate and lactate import in wt and *mfn1,2* muscle. In all panels, wt data are represented in gray, and *mfn1,2* data are represented in red. Box and whisker plots were plotted using the Tukey method. All data represent independent measurements from biological replicates. *P* values were calculated by multiple two-tailed *t* test (B, D, and G) with adjustments for multiple comparisons.

We next investigated the contribution of lactate as a carbon source, making use of steady-state infusion of [U-^13^C]lactate (Fig. 7, A and B). Lactate can be imported and oxidized within tissue (which we refer to as direct contribution); alternatively, extramuscular conversion of [U-^13^C]lactate to glucose m+3 can result in labeling in downstream metabolites via glycolysis (which we refer to as indirect contribution) (Fig. 7C). The labeling in glycolytic intermediates 3PG and PEP was significantly decreased in *mfn1,2* muscle, consistent with decreased indirect contribution from glycolysis (Fig. 7B). We normalized metabolite enrichments to labeling in 3PG or PEP to estimate the relative contribution of lactate via the direct pathway (Fig. 7D). After normalization, we observed significant increases in lactate’s contribution to pyruvate and TCA cycle intermediates in affected muscle (Fig. 7D). Thus, the perturbations in glycolysis in *mfn1,2* muscle correlate with an enhanced relative direct contribution of lactate to mitochondrial TCA cycle metabolites.

Direct measurements of net lactate usage in vivo are technically challenging in mice because of an inability to access the precise supply and draining vessels. Measurements of whole-body lactate turnover revealed that *mfn1,*2 animals displayed a trend toward higher circulatory turnover flux of lactate (fig. S6A), suggesting that there may be enhanced utilization of lactate by *mfn1,2* tissues. The precise source of circulating lactate in mammals is still under investigation; however, a recent report (*29*) indicates that slow-twitch muscles exhibit high usage of circulating glucose via glycolysis. We investigated the soleus muscle in wt and *mfn1,2* animals, which did not reveal changes in the levels of glycolytic enzymes, including the rate-limiting enzyme PFKM (fig. S6, B and C), or early glycolytic metabolites (fig. S6D). Pyruvate was significantly depleted in *mfn1,2* animals, while lactate tended to be up-regulated (fig. S6D). In addition, we observed depletion of a number of mitochondrial TCA cycle metabolites (fig. S6D) in the soleus of *mfn1,2* animals. Together, these results suggest that slow-twitch muscle may be trending toward increased glycolytic production of lactate in mutant animals.

To directly determine the relative contribution of lactate import in wt and *mfn1,2* muscle, we performed a steady-state infusion with [2-^2^H]lactate (Fig. 7, E and F). In this experiment, extramuscular exchange of lactate to pyruvate would result in loss of the deuterium label. Thus, observation of (m+1) lactate in tissue is a direct indicator of lactate import, and we observed an increased enrichment of (m+1) lactate in *mfn1,2* versus wt muscle (Fig. 7G). In wt animals, the muscle (m+1) lactate enrichment is ~44% of the plasma (m+1) lactate enrichment, indicating that ~44% of muscle lactate is derived from lactate import, while the other ~56% would be derived from intramuscular pyruvate (Fig. 7H). In *mfn1,2* animals, the muscle m+1 lactate enrichment is ~94% of the plasma (m+1) lactate enrichment, indicating that ~94% of muscle lactate is derived from lactate import, while the remaining ~6% is derived from intramuscular pyruvate (Fig. 7H). Thus, the altered glucose handling in *mfn1,2* muscle corresponds with a substantial increase in the relative direct contribution of lactate to intracellular metabolites. Intracellular conversion of lactate to pyruvate can result in transfer of the deuterium first to NADH and then to other potential metabolites including malate (Fig. 7E). We observed elevated levels of (m+1) malate in *mfn1,2* muscle (Fig. 7G), indicating a substantial contribution of lactate oxidation to cellular metabolism in diseased tissue. As a control, pyruvate and alanine are not expected to be labeled under these conditions, and correspondingly, we observed no changes in m+1 fractions of these metabolites (Fig. 7G).

### Glycolytic flux and AMPK status is regulated by a Hif1α-AMPD1 axis in *mfn1,2* muscle

**Fig. 8.**
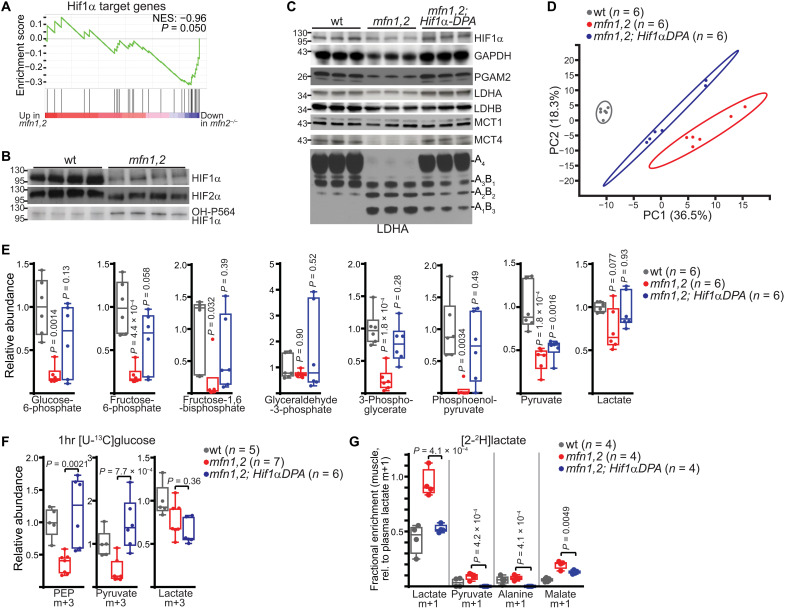
Reduced Hif1α slows glycolysis in *mfn1,2* muscle. (**A**) GSEA of Hif1α target genes in RNA-seq data from *mfn1,2* versus wt muscle. NES, normalized enrichment score; associated *P* value is indicated. (**B**) Hif1α and Hif2α protein levels, as assessed by Western blot, in wt and *mfn1,2* muscle. A Coomassie-stained blot is presented in Fig. 3D as a loading control. MW markers are indicated in kDa. (**C**) Levels of the indicated proteins (and LDH tetramer) in muscles from the indicated genotype. Coomassie-stained gels are presented in fig. S7E as a loading control, and quantitation of protein amounts is presented in fig. S7F. MW markers are indicated in kDa. (**D**) Principal components analysis of the metabolomes from TA muscle of the indicated genotype. The percentage of total variance for principal components 1 and 2 (PC1 and PC2) are indicated on the *x* and *y* axes. Ovals indicate the 95% confidence intervals. (**E**) Relative steady-state abundance of the indicated glycolytic metabolites. (**F**) Relative abundance of the indicated labeled species following a 1-hour [U-^13^C]glucose challenge. (**G**) Fractional enrichment of the indicated labeled species in muscle from steady-state [2-^2^H]lactate infusion, normalized to plasma lactate m+1 enrichment. Box and whisker plots were plotted using the Tukey method. All data represent independent measurements from biological replicates. *P* values were calculated by one-way ANOVA (E to G) with adjustments for multiple comparisons.

We turned our attention to the mechanism underlying the observed reduction in glucose utilization. A clue came from the relative perturbations of LDHA and MCT4 versus LDHB and MCT1 (Fig. 3, C and D, and fig. S7A). This led us to hypothesize a role for Hif1α whose established targets include LDHA and MCT4 (among other glycolytic genes), but not LDHB and MCT1 (*32*, *33*). Gene set enrichment analysis (GSEA) of our RNA-seq dataset indicated down-regulation of Hif1α targets (Fig. 8A), and Western blotting confirmed a notable loss of Hif1α protein in *mfn1,2* muscle and a modest loss of Hif2α protein (Fig. 8B). This was similar to the down-regulation of Hif1α target genes in the analyzed RIRCD patient dataset (Fig. 5F).

Hif1α transcripts were unaffected in *mfn1,2* tissue, indicating that loss of Hif1α is due to posttranslational mechanisms. The stabilization of Hif1α is known to be multifactorial in nature. Most prominently, Hif1α is stabilized in the setting of low oxygen; in addition, Hif1α can be stabilized by superoxide production (*34*, *35*). These phenomena result in inhibition of Hif1α PHD (prolyl hydroxylase), which normally hydroxylates Hif1α and targets the protein for subsequent degradation. Using a hydroxylation-specific antibody for Hif1α, we found that *mfn1,2* muscle exhibits increased levels of hydroxylated Hif1α (Fig. 8B), indicating that PHD activity is stimulated in *mfn1,2* muscle relative to wt muscle. To examine the reason for PHD stimulation, we investigated both reactive oxygen species (ROS) and oxygen status in vivo. *mfn1,2* tissue had a substantial reduction in ROS levels as reflected by a significant increase in the reduced to oxidized glutathione (GSH/GSSG) (fig. S7B). To examine oxygen status in vivo, we made use of a hypoxia-sensitive probe (pimonidazole), which allows identification of hypoxic regions in vivo (*36*). *mfn1,2* muscle exhibited significant reductions in staining intensity (fig. S7, C and D), indicating elevated oxygen levels in affected muscle. In particular, wt tissue displayed intense hypoxyprobe staining in subsarcolemmal regions of myofibers where nuclei and mitochondria accumulate, and this staining was absent in *mfn1,2* tissue (fig. S7D). On the basis of these results, we propose that the severe oxidative phosphorylation defects in *mfn1,2* muscle result in excess PHD activity due to elevated oxygen levels and decreased ROS, thereby resulting in a loss of Hif1α stability.

To assess the role of Hif1α loss in *mfn1,2* muscles, we reestablished Hif1α levels by introducing a conditional allele expressing the degradation-resistant Hif1α-dPA protein (*37*) into *mfn1,2* animals (hereafter *mfn1,2;Hif1*α*-dPA*). Reestablishing Hif1α levels rescued the levels of glycolytic enzymes in *mfn1,2* muscle (Fig. 8C and fig. S7, E and F), as well as shifted the LDH tetramer back to predominantly LDHA_4_ (Fig. 8C). Hif1α stabilization partially rescued metabolomes of *mfn1,2* muscle (Fig. 8D), including an increase in the abundance of glycolytic metabolites (Fig. 8E). Moreover, Hif1α stabilization was able to rescue contributions to glycolytic intermediates, measured with a 1-hour [U-^13^C]glucose challenge as above (Fig. 8F). Given the increased levels of LDHA and MCT4 in *mfn1,2;Hif1*α*-dPA* animals, we hypothesized that the relative lactate contribution to intracellular metabolites would be decreased. [2-^2^H]Lactate-labeling experiments revealed that reexpression of Hif1α lowers the relative contribution of imported lactate such that now ~53% of muscle lactate was derived from plasma import (similar to wt animals) (Fig. 8G).

**Fig. 9.**
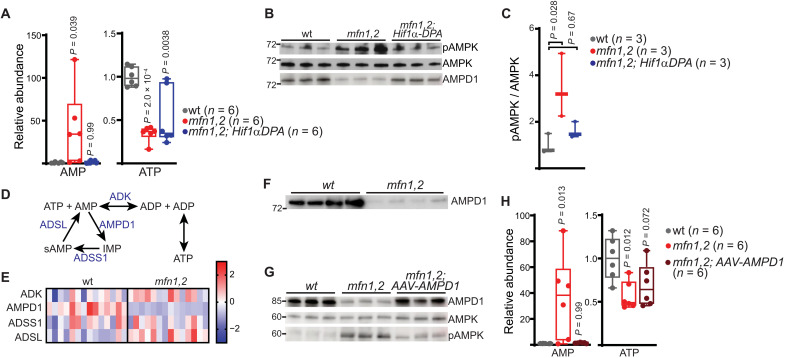
A Hif1α/AMPD1 axis regulates energy status in *mfn1,2* muscle. (**A**) Relative steady-state abundances of AMP and ATP in TA muscles of the indicated genotype. (**B**) Western blot for AMPK, phosphorylated AMPK (pAMPK), and AMPD1 in indicated TA muscle samples. A Coomassie-stained gel is presented in fig. S7E as a loading control. MW markers are indicated in kDa. (**C**) Quantitation of the relative ratios of pAMPK to AMPK. (**D**) Schematic of PNC; metabolites are indicated in black, and enzymes are indicated in blue. sAMP, adenylosuccinate; IMP, inosine monophosphate. (**E**) Heatmap showing *z* scores of protein abundances of the indicated genes in wt and *mfn1,2* muscle, based on proteomics analysis. *n* = 14 animals per genotype. (**F**) AMPD1 levels in wt and *mfn1,2* muscle. A Coomassie-stained blot is presented in Fig. 3D as a loading control. MW markers are indicated in kDa. (**G**) Western blot for AMPK, pAMPK, and AMPD1 in indicated TA muscle samples. A Coomassie-stained blot is presented in fig. S7H as a loading control. MW markers are indicated in kDa. (**H**) Relative steady-state abundances of AMP and ATP in the indicated TA muscle samples. Box and whisker plots were plotted using the Tukey method. All data represent independent measurements from biological replicates. *P* values were calculated by one-way ANOVA (A, C, and H) with adjustments for multiple comparisons.

As we observed increased contributions from glucose in the setting of Hif1α reintroduction, we probed energy status in *mfn1,2;Hif1*α*-dPA* muscle. The elevated levels of AMP in affected tissue were rescued by reexpression of Hif1α, despite a continued depletion of ATP (Fig. 9A). Correspondingly, the increased phosphorylation of AMPK was reverted in *mfn1,2;Hif1α-dPA* muscle (Fig. 9, B and C). These results suggested a role for Hif1α in regulating AMP levels outside its up-regulation of glycolysis-derived ATP. In principle, altered levels of any enzyme that consumes or produces AMP could regulate intracellular AMP concentration and AMPK status. In skeletal muscle, it has been proposed that the purine nucleotide cycle (PNC) consumes AMP to promote increased energy production by adenylate kinase (Fig. 9D) (*38*). We assessed levels of PNC enzymes in our proteomics dataset, which indicated a notable decrease in the levels of AMP deaminase (AMPD1) that was confirmed by Western blot, as well as a biochemical activity assay performed on muscle lysate (Fig. 9, E and F, and fig. S7G). AMPD1 levels were rescued by stabilization of Hif1α (Fig. 9B), suggesting that Hif1α directly stimulates production of AMPD1. Consistent with these data, an analysis of the AMPD1 promoter reveals a number of hypoxia response element (HRE) binding sites in the promoter regions in both human and rodent genomes (fig. S7I).

To test the sufficiency of AMPD1 to affect metabolism in *mfn1,2* muscle, we performed intramuscular injections of AAV (adeno-associated virus) expressing AMPD1 into the TA muscle of mutant mice, which was sufficient to reestablish AMPD1 levels and AMPD1 activity (Fig. 9G and fig. S7G). AAV-mediated expression of green fluorescent protein (GFP) or a catalytically inactive AMPD1 (AMPD1^D648G^) (*39*) did not restore AMPD1 activity (fig. S7G). Reexpression of AMPD1 was sufficient to lower AMP levels and inhibit phosphorylation of AMPK (Fig. 9, G and H). Thus, down-regulation of AMPD1 via Hif1α constitutes a key factor regulating the AMPK status of *mfn1,2* muscle.

### *mfn1,2* animals are dependent on lactate import for survival

**Fig. 10.**
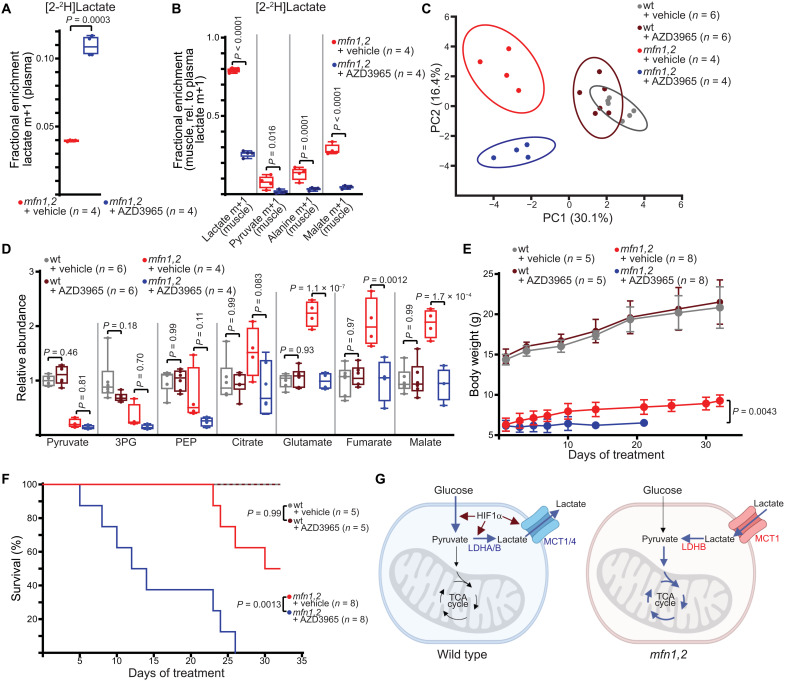
*mfn1,2* animals are dependent on lactate import. (**A**) Plasma labeling of lactate m+1 in steady-state infusion experiments with [2-^2^H]lactate in *mfn1,2* animals treated with vehicle or AZD3965. (**B**) Fractional enrichment of the indicated labeled species in *mfn1,2* muscle from steady-state [2-^2^H]lactate infusion, normalized to plasma lactate m+1 enrichment. (**C**) Principal components analysis of the metabolomes from TA muscle of the indicated genotype and treatment. The percentage of total variance for principal components 1 and 2 (PC1 and PC2) are indicated on the *x* and *y* axes. Ovals represent 95% confidence intervals. (**D**) Relative steady-state abundance of the indicated metabolites in TA muscle. (**E**) Longitudinal measurements of body weight in animals of the indicated genotype and treatment. (**F**) Fractional survival over time for animals of the indicated genotype and treatment. (**G**) Proposed model: In wt animals, Hif1α activity promotes expression of glycolytic genes, resulting in high levels of glucose utilization and lactate excretion. In *mfn1,2* animals, loss of Hif1α results in decreased glycolysis. As a result, lactate contribution to TCA cycle metabolites increases, and animals become dependent on lactate import via MCT1. Box and whisker plots were plotted using the Tukey method. All data represent independent measurements from biological replicates. *P* values were calculated by two-tailed *t* test (A), multiple two-tailed *t* test (B), two-way ANOVA (D and E), and Mantel-Cox test (F) with adjustments for multiple comparisons.

Lactate transport can be inhibited via an MCT1-specific inhibitor, AZD3965 (*40*). To verify AZD3965’s activity in *mfn1,2* animals in vivo, we performed [2-^2^H]lactate infusions in animals cotreated with AZD3965, which robustly inhibited lactate import and utilization in affected muscle such that only ~26% of muscle lactate was now derived from import (Fig. 10, A and B). We therefore treated *mfn1,2* animals daily with AZD3965 to assess the importance of lactate import in this disease model. A short-term treatment (3 days) altered the TA muscle metabolome of *mfn1,2* animals, but not wt animals (Fig. 10C). Glycolytic intermediates in *mfn1,2* muscle (including pyruvate and lactate) were largely unaffected by AZD3965 treatment; however, the levels of TCA cycle intermediates was significantly reduced in *mfn1,2* muscle by the inhibition of lactate import (Fig. 10D). These effects were not observed in wt littermates (Fig. 10D). Other organs were largely unaffected by AZD3965 treatment, although we did observe some changes in the levels of glycolytic intermediates (PEP, pyruvate, and lactate) in wt soleus and liver samples (fig. S8A). Together, these data implicate MCT1-mediated lactate import and utilization in *mfn1,2* TA muscle as being required for maintaining steady-state levels of TCA cycle intermediates.

To assess the effects on disease progression, we treated animals with daily administration of AZD3965 beginning at 3 weeks of age. The weight gain of AZD3965-treated *mfn1,2* animals immediately slowed, and their survival was rapidly compromised (Fig. 10, E and F). In contrast, treatments in wt littermates revealed no detrimental effects, consistent with the well-tolerated usage of this inhibitor in other studies (*30*). Thus, *mfn1,2* animals are sensitized to AZD3965 treatment, indicating that their survival and disease progression is dependent on lactate transport via MCT1.

## DISCUSSION

Our analysis of an early-onset animal model of MM revealed an unexpected metabolic finding: Glucose utilization by fast-twitch muscle is reduced in the setting of severe mitochondrial dysfunction (Fig. 10G). This result contrasts with the well-accepted notion of enhanced glycolysis in the setting of mitochondrial deficits, which is largely based on cultured studies. Classically, patients with severe mitochondrial dysfunction and neurological involvement have exhibited lactic acidosis, particularly in the settings of MELAS (mitochondrial encephalopathy, lactic acidosis, and stroke-like episodes) syndrome and Leigh syndrome, and this clinical finding can be representative of increased glycolytic flux (*41*). However, lactic acidosis is not a universal finding in mitochondrial diseases; large-scale studies indicate that only 30 to 50% of patients with mitochondrial disorders have an elevated blood lactate level (*42*, *43*).

While decreased glycolytic activity could be due to a number of reasons, including impaired recycling of NADH to NAD^+^ in the setting of complex I dysfunction, our results indicate that constitutive Hif1α signaling is required to both maintain glycolysis and regulate AMPK activation in severe MM. In particular, a number of Hif1α-regulated transcripts are down-regulated in our animal model and patient biopsy samples. Some “glycolytic” enzymes are not transcriptionally regulated by Hif1α, including GLUT4, LDHB, and MCT1, and these genes were unaffected or up-regulated in our analysis. MCT1 and LDHB likely promote lactate import and oxidation, while GLUT4 and HK2 may allow glucose entry for alternative fates, including serine/glycine synthesis and the pentose phosphate pathway. Thus, a surprising role for mitochondrial ETC function in fast-twitch muscle is to control glucose utilization via regulation of Hif1α levels, potentially via alterations in oxygen or ROS levels. Consistent with previous studies, we also observed AMPK activation in this in vivo setting of mitochondrial dysfunction. However, AMPK activation in severe MM is not directly regulated by a loss of mitochondrial function in this model; instead, AMPK activity is tied to the PNC via Hif1α-dependent expression of AMPD1.

The loss of glycolysis in severe MM induces a notable dependence on lactate import (Fig. 10G), an unexpected nutrient dependency that regulates disease progression. Although lactate has been commonly considered a waste product based on in vitro experimentation, it has been appreciated that in vivo lactate constitutes a substantial carbon source for some tissues (*29*, *44*). The precise roles for lactate utilization in vivo in wt animals are still under intense investigation, but the relevance is difficult to formally test as the lactate transporters and processing enzymes (e.g., LDHA, LDHB, MCT1, and MCT4) can display bidirectional activity. In vitro, biochemical analysis suggest that LDHA prefers conversion of pyruvate to lactate, based on an increased affinity for pyruvate, while LDHB operates in the reverse direction (*45*). Similarly, MCT1’s high affinity for lactate suggests that it primarily imports lactate, while MCT4 serves as lactate exporter (*46*). Our *mfn1,2* model, in which LDHA and MCT4 are down-regulated, exhibits increased contribution from lactate import and suggests that the in vivo functions of these enzymes are generally consistent with the measured biochemical parameters. A limitation of this study is that the genetic perturbations in our animal model are limited to fast-twitch muscle fibers, which can have activity-dependent changes in metabolism. These data are largely focused on experiments in sedentary animals, and it will be interesting in the future to examine the metabolic response during various forms of exercise. In addition, an analysis of the metabolic perturbations induced in slow-twitch muscle will be of interest, although a slow-twitch specific Cre driver has yet to be developed. However, we should emphasize that fast-twitch muscle is a common site of pathology and biopsy in early-onset myopathy patients. Thus, our findings emphasize a role for lactate utilization in regulating disease progression and may therefore inform on potential nutrient strategies for patients suffering from early-onset forms of MM.

## MATERIALS AND METHODS

### Animals

All mouse experiments were performed according to protocols approved by the Institutional Animal Care and Use Committee at the University of Texas Southwestern Medical Center (protocol 101597). Experimental and wt littermates were generated as previously described (*19*) by crossing Myl1-Cre;Mfn1^+/−^;Mfn2^+/−^ mice to mDendra^flox/flox^; Mfn1^flox/flox^; Mfn2^flox/flox^ mice. “*mfn1,2*” pups were identified by genotyping as Myl1-Cre; Mfn1^−/flox^; Mfn2^−/flox^, and “wt” pups were identified by genotyping as Mfn1^+/flox^; Mfn2^+/flox^. For introduction of the Hif1αDPA allele, *mfn1,2;Hif1aDPA* mice (Myl1-Cre; Hif1αDPA^flox/flox^; Mfn1^−/flox^; Mfn2^−/flox^) were generated by crossing Myl1-Cre; Hif1dPA^flox/flox^; Mfn1^+/−^; Mfn2^+/−^ mice to Hif1dPA^flox/flox^; Mfn1^flox/flox^; Mfn2^flox/flox^ mice. “*mfn1,2;Hif1αDPA*” pups were identified by genotyping as Myl1-Cre; Hif1aDPA^flox/flox^; Mfn1^−/flox^; Mfn2^−/flox^. Mice were housed on standard chow in small groups on a 12-hour light/12-hour dark cycle. All mice were analyzed at 4 weeks of age, unless otherwise indicated. For transcriptomics, proteomics, and metabolomic screening experiments in Fig. 3, we used age-matched littermates from 4 to 10 weeks of age. Both male and female mice were used in all experiments; where sex-specific differences were not present, male and female mice were analyzed together. For AZD3965 treatments, 4-week-old wt and *mfn1,2* littermates were treated with the MCT1 inhibitor AZD3965 by oral gavage every day (30 mg kg^−1^ body mass in 100 μl of 0.5% promethylcellulose, 0.2% Tween 80, and 5% dimethyl sulfoxide) based on a previous protocol (*30*).

Mice forelimb and hindlimb grip strength was measured by blinded staff at the UT Southwestern Neuro-Models Facility using a wire mesh grid connected to a horizontally aligned force meter (San Diego Instruments). Mice were lifted by their tail and moved toward the top rung of the grid until they tightly gripped the bar with their forelimbs or hindlimbs. Mice were gently pulled away from the bar in a straight line until the grasp was broken and the peak force measurement was recorded. The forelimbs and hindlimbs were tested separately; for each, six trials were repeated per mouse, and the low values were discarded. The average of the remaining force measurements was normalized to body weight.

For treadmill experiments, mice were acclimated to the treadmill (Columbus Instruments) by running 30 min per day for 3 days at slow speeds (1 to 5 m/min). On the fourth day, mice were run on the treadmill for 30 min at 10 m/min. Mice were immediately anesthetized, and TA muscles were rapidly dissected and flash-frozen in liquid nitrogen.

For insulin and glucagon measurements, mice were fasted overnight, and insulin and glucagon were measured in plasma samples using the Ultra Sensitive Mouse Insulin ELISA Kit (Crystal Chem, 90080) or the Mouse Glucagon ELISA Kit (Crystal Chem, 81518), following the manufacturer’s instructions. For glucose tolerance testing, fasted mice were gavaged with d-glucose (2 g/kg body weight). Plasma glucose levels were measured from tail vein blood using a glucose monitor (Contour). For insulin tolerance testing, fasted mice were intraperitoneally injected with Humulin R Insulin [0.5 U/kg diluted in saline + 0.1% bovine serum albumin (BSA); Fisher Scientific, NC1415864], followed by plasma glucose measurements as above.

### Oxygen consumption measurements

Isolated mitochondria from TA muscles were prepared by differential centrifugation as previously described (*47*). Oxygen consumption measurements were performed in a Seahorse XFe96 instrument based on a previously published protocol (*48*). Briefly, 1 μg of isolated mitochondria was plated per well in mitochondrial isolation buffer [10 mM Hepes, 70 mM sucrose, 220 mM mannitol, 1 mM EGTA, and 5 mM MgCl_2_ (pH7.2)], followed by supplementation with isolation buffer + 10 mM glutamate and 2 mM malate. Each measurement was performed for 3 min at 37°C after a 3-min mixing period. Sequential injections of ADP (final concentration, 4 mM), oligomycin (final concentration, 2 μM), carbonyl cyanide 3-chlorophenylhydrazone (CCCP) (final concentration, 1 μM), and antimycin (final concentration, 1 μM) were performed. State 3 (following ADP injection) and state 4o (following oligomycin injection) were calculated after baseline subtraction (from wells with no mitochondria). The respiratory control ratio was calculated as the state 3 respiration divided by the state 4o respiration.

### Histologic analysis of muscle tissue

TA muscles were rapidly dissected under anesthesia and then immediately frozen in liquid nitrogen–cooled 2-methylbutane (Sigma-Aldrich, M32631) before being embedded into an optimal cutting temperature compound (Thermo Fisher Scientific, 23-730-571). Sections (10 μm) were cut on a cryostat (Leica, CM3050S). For COX and SDH activity staining, slides were stained based on standard operating protocols (https://neuromuscular.wustl.edu/pathol/histol/cox.htm; https://neuromuscular.wustl.edu/pathol/histol/SDH.pdf) and imaged using an Olympus IX83 microscope. For fiber-type analysis, sections were blocked with 10% goat serum (Fisher Scientific, 16-210-064) in PBST [0.25% Triton X-100 in phosphate-buffered saline (PBS)] for 1 hour at room temperature, followed by incubation with anti–myosin heavy chain type I (BA-D5, Developmental Studies Hybridoma Bank), anti–myosin heavy chain type IIa (SC-71, Developmental Studies Hybridoma Bank), anti–myosin heavy chain type IIb (BF-F3, Developmental Studies Hybridoma Bank), and anti-laminin (Sigma-Aldrich, L9393) antibodies overnight at 4°C. Alexa Fluor 594 goat anti-mouse immunoglobulin G1 (IgG1) (Invitrogen, A21125), Alexa Fluor 647 goat anti-mouse IgG2b (Invitrogen, A21242), Alexa Fluor 488 goat anti-rabbit IgG (H+L) (Invitrogen, A11034), and DyLight 405 goat anti-mouse IgM (Jackson ImmunoResearch, 115-474-075) secondary antibodies were then added for 1 hour at room temperature before the slides were mounted and imaged using a Zeiss Axioscan Z1 microscope.

Hypoxia in muscles of wt and *mfn1,2* mice were detected using the Hypoxyprobe-Red549 Kit (HPI, HP7-XXX) according to the manufacturer’s instructions. Mice were administered with Hypoxyprobe (Hypoxyprobe-1; 60 mg/kg) via retro-orbital injection. TA muscles were harvested and frozen after 90 min. Cryosections (10 μm) were fixed with 4% paraformaldehyde (10 min) and then were blocked with 10% goat serum (Fisher Scientific, 16-210-064) in PBST (0.25% Triton X-100 in PBS) for 1 hour at room temperature, followed by incubation with mouse anti-pimonidazole antibody conjugated to DyLight 549 overnight at 4°C. Cryosections were then rinsed with PBS and incubated with DAPI (4′,6-diamidino-2-phenylindole) (Sigma-Aldrich, D9542) and Alexa Fluor 488–conjugated Wheat Germ Agglutinin (Thermo Fisher Scientific, W11261). Slides were scanned with an LSM 880 confocal microscope (Zeiss).

### In situ muscle force measurements

TA muscle force was measured using the following protocol (http://treat-nmd.eu/downloads/file/sops/dmd/MDX/DMD_M.2.2.005.pdf) in animals at 4 weeks of age. Mice were anesthetized with isoflurane, and the sciatic nerve was exposed in the posterolateral thigh and clamped to a custom electrode. The distal tendon of the TA muscle was exposed, severed, and attached to a force transducer via a suture (Grass Instruments, #FT03-E). During measurement, the TA muscle and nerve were kept moist with 37°C saline. The TA muscle was stimulated via the sciatic nerve using a pulse generator (Siglent Technologies, #SDG2042X). A digital acquisition board (DATAQ Instruments, #DI-1110) recorded the force output using WinDaq software (v.3.0.7). The muscle length was optimized to achieve maximal force, followed by optimization of supramaximal stimulation voltage (typically 3 to 4 V) and pulse duration (typically 0.3 to 0.4 ms). Maximal twitch force was collected for five to six trials and was normalized by muscle weight. Following measurement of twitch force, the muscle was stimulated at 120 Hz at supramaximal stimulation to collect tetanic force output. Data were analyzed and plotted in MATLAB (MathWorks Inc.).

### Ex vivo muscle glycolysis experiments

TA muscles were rapidly dissected from mice under anesthesia and immediately transferred to six-well plates containing 2 ml of glucose-free Dulbecco’s modified Eagle’s medium (Thermo Fisher Scientific, 11966025) supplemented with 10 mM [1,6-^13^C]glucose (Cambridge Isotope Laboratories, CLM-2717). At specific time points (0, 10, 30, 60, 120, and 180 min), 20 μl of medium was removed and added to 1 ml of 80% MeOH spiked with 200 nmol of [U-^13^C]lactate. Samples were vortexed and centrifuged at 13,000*g* for 10 min at 4°C. The supernatant was collected and dried down by SpeedVac, followed by derivatization and analysis by gas chromatography–mass spectrometry (GC-MS) (detailed below). To calculate the nanomoles of m+1 lactate present in the medium sample, the m+1 lactate peak area was normalized to the area of the spiked m+3 lactate peak.

### In vivo isotope tracing

All in vivo isotope tracing experiments were performed in conscious mice after overnight fasting using previously published protocols that did not disturb plasma levels of each infused metabolite (*24*, *27*, *28*, *30*). On the morning of the experiment, catheters (Braintree Scientific, MRE-KIT 025) were surgically implanted into the right external jugular vein of mice. For steady-state euglycemic infusion of [U-^13^C]glucose, a total dose of 8 g/kg body mass (dissolved in 1000 μl of saline) was continuously infused at 2.5 μl min^−1^ (total infusion time of 5 hours). For measurements of F_circ_glucose, [U-^13^C]glucose was infused at 3.6 mg min^−1^ kg^−1^ (0.1 μl min^−1^ g^−1^ of a 200 mM solution) for 2.5 hours. For steady-state infusion of [^13^C3,^15^N]alanine, a total dose of 162 mg/kg alanine was continuously infused at 0.05 μl min^−1^ g^−1^ over 3 hours. For steady-state infusion of [U-^13^C]lactate, a total dose of 1.44 g/kg dissolved in 750 μl of saline was infused over 10 min in 150 μl of saline, followed by a continuous infusion of 0.0048 mg/g body mass per minute for 3 hours in 360 μl of saline. For steady-state infusion of [2-^2^H]lactate, a total dose of 1.44 g/kg dissolved in 750 μl of saline was continuously infused at 2 μl/min over 4 hours. For infusions in the presence of AZD3965, a working solution of AZD3965 was first made (200 μM AZD3965 in 950 μl of saline and 50 μl of βcyclodextrin), and the total dose of 1.44 g/kg was dissolved in 750 μl of the working solution. At the end of the infusion, mice were anesthetized and tissue was immediately harvested, snap-frozen in liquid N_2_, and stored at −80°C before GC-MS analysis. For short-term glucose utilization measurements, a single dose of 2 g/kg body mass [U-^13^C]glucose was delivered by oral gavage, mice were anesthetized, and tissue was harvested for analysis 1 hour later.

### Metabolite MS analysis

Isotope enrichments in glycolytic and TCA cycle metabolites were quantitated by GC-MS. Muscle samples were homogenized using an electronic tissue disruptor (Qiagen) in ice-cold 80:20 methanol:water (v/v), followed by three freeze-thaw cycles in liquid nitrogen. The supernatant was collected after a 10 min centrifugation at 13,000*g* at 4°C and dried down overnight in a SpeedVac instrument. For plasma and medium analysis, 20 to 40 μl of plasma or medium were added to 80:20 methanol:water and then dried down using SpeedVac (Thermo Fisher Scientific). Dried metabolites were derivatized to form methoxime-tert-butyldimehtylsilyl (TBDMS) adducts by incubating with 1% methoxyamine hydrochloride (Sigma-Aldrich) in pyridine at 70°C for 15 min, followed by the addition of *N*-*tert*-butyldimethylsiyl-*N*-methyltrifluoroacetamide (Sigma-Aldrich, 1014) for 1 hour. One microliter of aliquots was injected for analysis. Samples were analyzed using either an Agilent 6890 or an Agilent 7890 gas chromatograph coupled to an Agilent 5973N or 5975C Mass Selective Detector, respectively. The observed distributions of mass isotopologs were corrected for natural abundance (*49*).

For liquid chromatography–tandem mass spectrometry (LC-MS/MS) analysis of steady-state metabolite levels, muscle samples (~20 mg) were pulverized with a mortar and pestle on liquid nitrogen (H37260-0100, Bel-Art Products), collected in 80% methanol, and subjected to three freeze-thaw cycles in liquid nitrogen. For plasma analysis, 20 μl of samples was extracted with 0.2 ml of cold acetone. Protein precipitates were removed by centrifugation. Data acquisition was performed by reversed-phase chromatography on a 1290 UHPLC LC system interfaced to a high-resolution MS 6550 iFunnel Q-TOF mass spectrometer (Agilent Technologies, CA). The MS was operated in both positive and negative [electrospray ionization (ESI)^+^ and ESI^−^] modes. Analytes were separated on an Acquity UPLC HSS T3 column (1.8 μm, 2.1 mm × 150 mm; Waters, MA). The column was kept at room temperature. Mobile phase A composition was 0.1% formic acid in water, and mobile phase B composition was 0.1% formic acid in 100% acetonitrile (ACN). The LC gradient was 0 min: 1% B; 5 min: 5% B; 15 min: 99% B; 23 min: 99% B; 24 min: 1% B; and 25 min: 1% B. The flow rate was 250 μl min^−1^. The sample injection volume was 5 μl. ESI source conditions were set as follows: dry gas temperature of 225°C and flow of 18 liters min^−1^, fragmentor voltage of 175 V, sheath gas temperature of 350°C and flow of 12 liters min^−1^, nozzle voltage of 500 V, and capillary voltage of +3500 V in positive mode and −3500 V in negative mode. The instrument was set to acquire over the full *m*/*z* (mass/charge ratio) range of 40 to 1700 in both modes, with the MS acquisition rate of 1 spectrum s^−1^ in profile format. Raw data files were processed using Profinder B.08.00 SP3 software (Agilent Technologies, CA) with an in-house database containing retention time and accurate mass information on 600 standards from Mass Spectrometry Metabolite Library (IROA Technologies, MA), which was created under the same analysis conditions. The in-house database matching parameters were mass tolerance of 10 parts per million (ppm) and retention time tolerance of 0.5 min. Peak integration result was manually curated in Profinder. Metabolite abundances were normalized by total ion count (TIC) for each sample. Principal components analysis was performed using MATLAB (MathWorks).

NAD(P)^+^/NAD(P)H was measured as previously described (*18*). Briefly, ~20 mg of tissue was homogenized on ice in cold 40:40:20 (ACN:methanol:water) with 0.1 M formic acid buffer. The samples were vortexed, chilled, quenched with 15% ammonium bicarbonate, and centrifuged to pellet protein precipitates. Supernatants were diluted in 10 mM ammonium bicarbonate, and ^15^N_5_-AMP (Sigma-Aldrich, 662658) was added as an internal standard and injected onto a Sciex 6500 Qtrap using a reversed-phase ion pairing method. Pelleted protein was lysed and quantified using the DC protein assay (Bio-Rad 5000112).

### Proteomics analysis of muscle tissue

Thirty micrograms of muscle protein homogenates was solubilized in 1× SDS buffer and then loaded onto a 4 to 15% Mini-PROTEAN TGX precast protein gel, followed by SDS–polyacrylamide gel electrophoresis (SDS-PAGE) separation. The gels were stained with Coomassie Blue, and gel slices were excised into 1 mm^3^ cubes. Following procedures previously described in (*50*), gel slices were digested overnight with trypsin (Pierce, 90058), following reduction and alkylation with dithiothreitol and iodoacetamide (Sigma-Aldrich, I6125). The samples then underwent solid-phase extraction cleanup with an Oasis HLB plate (Waters), and the resulting samples were injected onto an Orbitrap Fusion Lumos mass spectrometer coupled to an Ultimate 3000 RSLC-Nano LC system. Samples were injected onto a 75-μm-internal-diameter, 75-cm-long EasySpray column (Thermo Fisher Scientific) and eluted with a gradient from 0 to 28% buffer B over 90 min. Buffer A contained 2% (v/v) ACN and 0.1% formic acid in water, and buffer B contained 80% (v/v) ACN, 10% (v/v) trifluoroethanol, and 0.1% formic acid in water. The mass spectrometer was operated in a positive ion mode with a source voltage of 1.8 kV and an ion transfer tube temperature of 275°C. MS scans were acquired at 120,000 resolution in the Orbitrap, and up to 10 MS/MS spectra were obtained in the ion trap for each full spectrum acquired using higher-energy collisional dissociation for ions with charges 2 to 7. Dynamic exclusion was set for 25 s after an ion was selected for fragmentation. Raw MS data files were analyzed using Proteome Discoverer v2.4 SP1 (Thermo Fisher Scientific), with peptide identification performed using Sequest HT searching against the mouse protein database from UniProt. Fragment and precursor tolerances of 10 ppm and 0.6 Da were specified, and three missed cleavages were allowed. Carbamidomethylation of Cys was set as a fixed modification, with oxidation of Met set as a variable modification. The false discovery rate (FDR) cutoff was 1% for all peptides. Protein abundances were normalized by TIC. Unsupervised hierarchical clustering was performed using MATLAB (MathWorks), and statistical significance was assessed by multiple *t* tests (GraphPad Prism). Gene ontology analysis was performed using DAVID software (https://david.ncifcrf.gov/).

### DNA isolation and analysis

Frozen muscle samples were homogenized with a handheld homogenizer in very short bursts, and total DNA was purified using phenol-chloroform extraction. The mtDNA copy number was quantitated by quantitative PCR (qPCR) using primers specific to mouse mtDNA and nuclear DNA (nDNA), and the ratio was calculated using the 2^−ΔΔCT^ method (*51*). Long-range PCR over the mitochondrial genome was performed using the Expand Long Template PCR system (Sigma-Aldrich, 11681834001), following the manufacturer’s instructions. Samples were visualized on a 0.5% agarose gel.

### RNA isolation and RT-qPCR analysis

Frozen muscle samples were homogenized with a handheld homogenizer in very short bursts. Total RNA was isolated using the RNeasy Fibrous Tissue Mini Kit (QIAGEN, 74704). Reverse transcription qPCR (RT-qPCR) was performed in duplicate with the Luna Universal One-Step RT-qPCR Kit (New England Biolabs, E3005L) using the CFX384 Touch Real-Time PCR Detection System (Bio-Rad Laboratories). The mRNA level in each sample was normalized to the level of the Actinb gene transcript, and amplification specificity was assessed using melting curve analysis. Relative expression levels were calculated using the 2^−ΔΔCt^ method (*51*).

### RNA-seq and data analysis

The RNA-seq library was prepared using the SMARTer Stranded Total RNA Sample Prep Kit (Takara Bio) following the manufacturer’s protocol. Amplified libraries were purified using AMPure XP beads (Beckman Coulter), quantified, and sequenced (1 × 75 base pairs) on an Illumina NextSeq 500 system. Sequencing analysis was performed using the BICF RNASeq Analysis Workflow (ssh://git@git.biohpc.swmed.edu/BICF/Astrocyte/rnaseq.git). Normalized transcript values and statistical analysis were calculated using DESEQ2. Gene ontology analysis was performed using DAVID software (https://david.ncifcrf.gov/).

### LDH isoenzyme characterization

Muscle lysates were homogenized in liquid nitrogen and then resuspended in lysis buffer from the Lactate Dehydrogenase Staining Kit (Biomedical Research Service Center E-106) and loaded onto 7.5% Native gel (Bio-Rad, 4561023). Electrophoresis was conducted for 120 min at 120 V in 5 mM tris-HCl and 40 mM glycine (pH 9.5) running buffer. Gels were then transferred to polyvinylidene difluoride (PVDF) membranes via semidry transfer and blotted with an antibody against LDHA (Proteintech, 21799-1-AP).

### AAV intramuscular infections

Complementary DNAs (cDNAs) for murine AMPD1, AMPD1^D648G^, or GFP were cloned in the px700 vector (modified from Addgene 85741 by replacing the U6-EF1α-EYFP region with a CMB promoter). AAVpro cells (8 × 10^6^) (Takara Bio, 632273) were transfected with 12 μg of pAdDeltaF6 (Addgene, 112867), 6 μg of pAAV2/9n (Addgene, 112865), and 6 μg of pX700 using PEI MAX (Polysciences, 24765-1). Viral supernatant was collected 4 days after transfection, purified by ultracentrifugation using an iodixanol gradient, and then concentrated using a 100-kDa ultracentrifugal filter (Amicon UFC810024). AAV titers were quantified by qPCR. Intramuscular injection of *mfn1,2* mice was performed at 3 weeks of age using 10 μl of solution at a total dose of 5 × 10^10^ viral genomes. Tissue was harvested 3 weeks after injection for analysis.

### AMPD activity assay

TA muscle lysates were subjected to two rounds of freeze-thaw cycle, resuspended in ice-cold deionized water, and used directly for quantification of AMPD activity, according to the manufacturer’s instructions (Creative BIOMART, kit 0875). The enzymatic activity of AMPD1 was monitored at 340 nm every 1 min at 37°C for 1 hour using a SpectraMax iD3 plate reader (Molecular Devices).

### Western blot analysis

Protein lysates were homogenized in liquid nitrogen, resuspended in radioimmunoprecipitation assay buffer (Thermo Fisher Scientific, 89901), and run on precast SDS-PAGE gels (Bio-Rad, 4561093DC). The gels were then transferred to PVDF membranes (Millipore, 4729) using a semidry transfer system (Bio-Rad, 1704150) in transfer buffer [25 mM tris and 192 mM glycine (pH8.3) with 20% methanol (v/v)]. Membranes were blocked in 5% (w/v) BSA in Tris-buffered saline with 0.1% Tween 20 (TBS-T) buffer for 1 hour. Primary antibodies were incubated with membranes for 2 hours at room temperature, washed three times with TBS-T buffer, and then incubated with secondary antibody for 1 hour at room temperature. Membranes were incubated with ECL Western HRP substrate (Millipore, WBKLS0500) and detected using an Amersham ImageQuant 800 instrument. Because of the large number of protein changes between the two genotypes, we chose to normalize the Western blot intensity by total protein amounts (*52*) as follows: After immunodetection, membranes were washed with TBS-T and stained using the Pierce MemCode Reversible Protein Stain Kit for Western blotting (Thermo Fisher Scientific, 24585) according to the manufacturer’s instructions. Alternatively, gels were stained for total protein using the Colloidal Blue Staining Kit (Thermo Fisher Scientific, LC6025). Membranes/gels were scanned using an Amersham ImageQuant 800 instrument, and staining intensity for each lane was calculated using ImageJ. The following antibodies were used: MCT1 (Proteintech, 20139-1-AP), MCT4 (Proteintech, 22787-1-AP), LDHA (Proteintech, 21799-1-AP), LDHB (Proteintech, 14824-1-AP), AMPD1 (Proteintech, 19780-1-AP), AMPK (Cell Signaling Technology, 2532S), pAMPK (Cell Signaling Technology, 2535 T), GAPDH (Proteintech, HRP-60004), HIF1α (Novus Biologicals, NB100-479), Hif2α (Novus Biologicals, NB100-122), OH-P564 HIF1α (Cell Signaling Technology, 3434S), PGAM2 (Proteintech, 15550-1-AP), Tomm20 (11802-1-AP), PFKM (Proteintech, 550281-1-AP), PGM1 (Proteintech, 15161-AP), PHKA1 (Proteintech 24279-1-AP), and PHKG2 (Proteintech, 151091-AP).

### Analysis of human biopsy sample data

Microarray data from four TK2-deficient patients and three controls collected by Kalko *et al*. (*25*) were downloaded from the National Center for Biotechnology Information (NCBI) Gene Expression Omnibus (GEO) (accession GSE43698) and analyzed using GEO2R (https://ncbi.nlm.nih.gov/geo/geo2r/) to calculate fold changes and *P* values (reported in table S3). Significantly down-regulated proteins were identified by log_2_(fold change) < −1 and *P* < 0.05. Gene ontology analysis for Biological Processes (BP direct) was performed using DAVID software (https://david.ncifcrf.gov/).

RNA-seq data from five RIRCD patient muscle samples and six control muscle samples and proteomics data from three RIRCD muscles and three control muscles collected by Hathazi *et al*. (*26*) were directly downloaded at https://doi.org/10.15252/embj.2020105364. Significantly down-regulated transcripts or proteins were identified by log_2_(fold change) < −1 and adjusted *P* < 0.05. Gene ontology analysis for Biological Processes (BP) was performed using DAVID software (https://david.ncifcrf.gov/).

### Statistical analysis

All data are from biological replicates and represented either as mean and SDs or via box and whisker plots using the Tukey method. No statistical tests were used to predetermine sample size. Animals were randomly allocated into experimental groups. No blinding or masking of samples was performed. Datasets for each group of measurement were tested for normality using the Shapiro-Wilk test. If the data were not normally distributed, the data were log-transformed and retested for normality. For normally distributed data, groups were compared using the two-tailed Student’s *t* test (for two groups) or one-way analysis of variance (ANOVA) or two-way ANOVA (>2 groups), followed by Tukey’s or Dunnett’s test for multiple comparisons. For data that were not normally distributed, we used nonparametric testing (Mann-Whitney or Kolmogorov-Smirnov tests for two groups and Kruskal-Wallis test for multiple groups), followed by Dunn’s multiple comparisons adjustment. For metabolomics and proteomics datasets, abundances were compared by multiple *t* tests, followed by Bonferroni correction of *P* values. Proteomics and metabolomics analysis were performed once using biological replicates (individual mice); for other experiments, multiple (two to four) independent experiments with biological replicates (individual mice) were performed for reported data, and the number of biological replicates is indicated in the figures. No data were excluded.

## Notes

View/request a protocol for this paper from *Bio-protocol*.
