# Supplementary material for: Lactate metabolism is essential in early-onset mitochondrial myopathy
Source: Sci Adv. 2023 Jan 4;9(1):eadd3216. doi: 10.1126/sciadv.add3216 (PMC9812384; doi:10.1126/sciadv.add3216)
Supplement: Supplementary file 1 — Figs. S1 to S8 [file sciadv.add3216_sm.pdf]

Science Advances
AAAS

Supplementary Materials for

Lactate metabolism is essential in early-onset mitochondrial myopathy

Zhenkang Chen *et al.*

Corresponding author: Prashant Mishra, prashant.mishra@utsouthwestern.edu

Sci. Adv. **9**, eadd3216 (2023)
DOI: 10.1126/sciadv.add3216

The PDF file includes:

Figs. S1 to S8
Legends for tables S1 to S3

Other Supplementary Material for this manuscript includes the following:

Tables S1 to S3

Figure S1

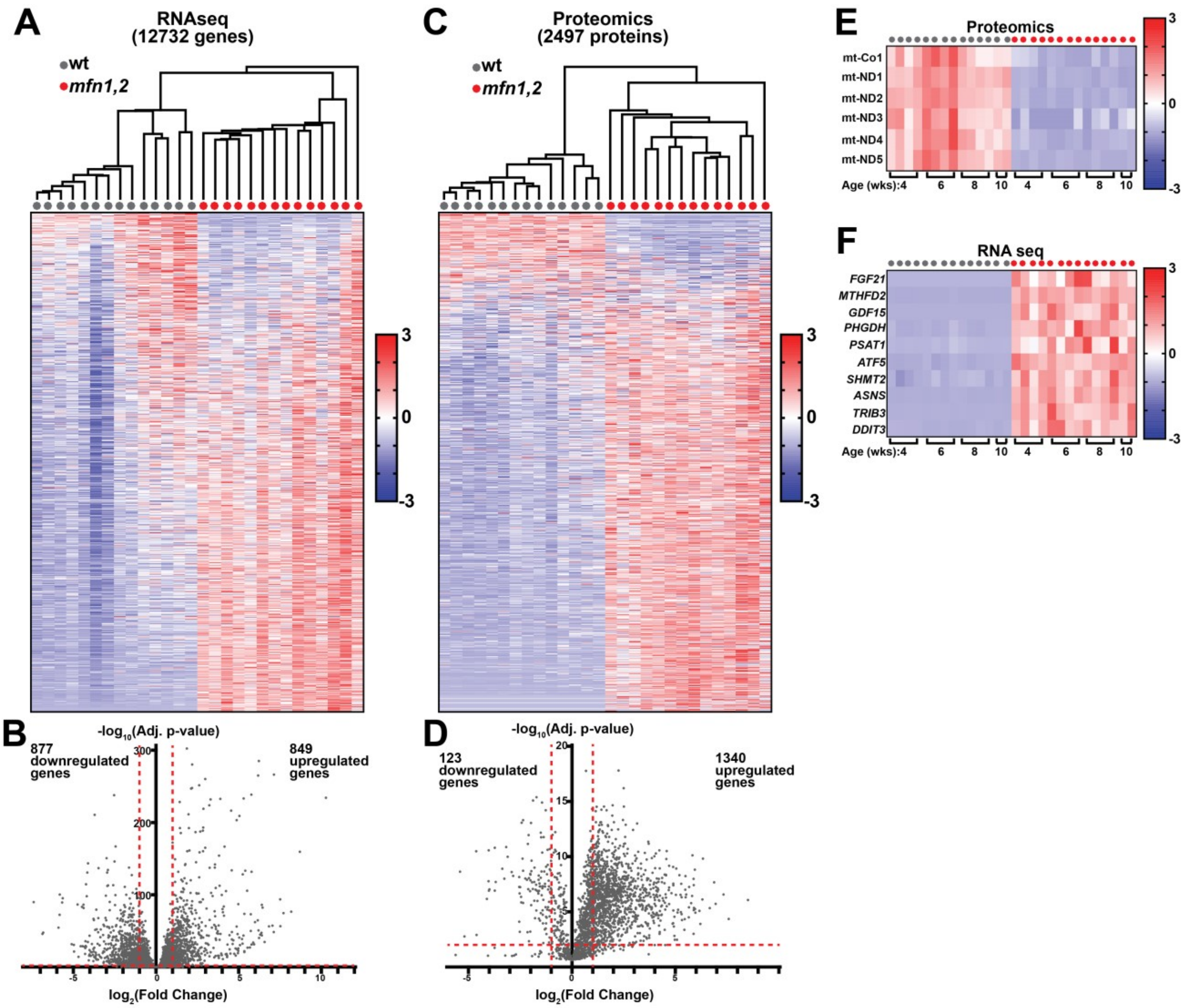

Fig. S1. RNAseq and proteomic differences in wild-type and *mfn1,2* muscle. (A) Unsupervised hierarchical clustering of normalized RNAseq values of transcripts in wt and *mfn1,2* muscle. Heatmaps of z-scores are presented. n=14 animals per genotype. **(B)** Volcano plot of gene expression changes in *mfn1,2* vs. wt muscle, based on RNAseq analysis. Log_2(Fold Change) is plotted against the $-log_{10}$(adjusted p-value) for each gene. The numbers of significantly upregulated and downregulated genes are indicated. **(C)** Unsupervised hierarchical clustering of normalized proteomics values of individual proteins in wt and *mfn1,2* muscle. Heatmaps of z-scores are presented. n=14 animals per

genotype. **(D)** Volcano plot of protein abundance changes in *mfn1,2* vs. wt muscle, based on RNAseq analysis. Log_2(Fold Change) is plotted against the $-log_{10}$(adjusted p-value) for each gene. The numbers of significantly upregulated and downregulated genes are indicated. **(E)** Protein abundances for mtDNA-encoded genes in wild-type and *mfn1,2* muscle of indicated ages are presented as a heatmap of z-scores. **(F)** Transcript abundances for ISR^{mt} genes in wild-type and *mfn1,2* muscle of indicated ages are presented as a heatmap of z-scores. All data represent independent measurements from biological replicates. P-values were calculated by multiple two-tailed t-tests (B,D) with adjustments for multiple comparisons.

Figure S2

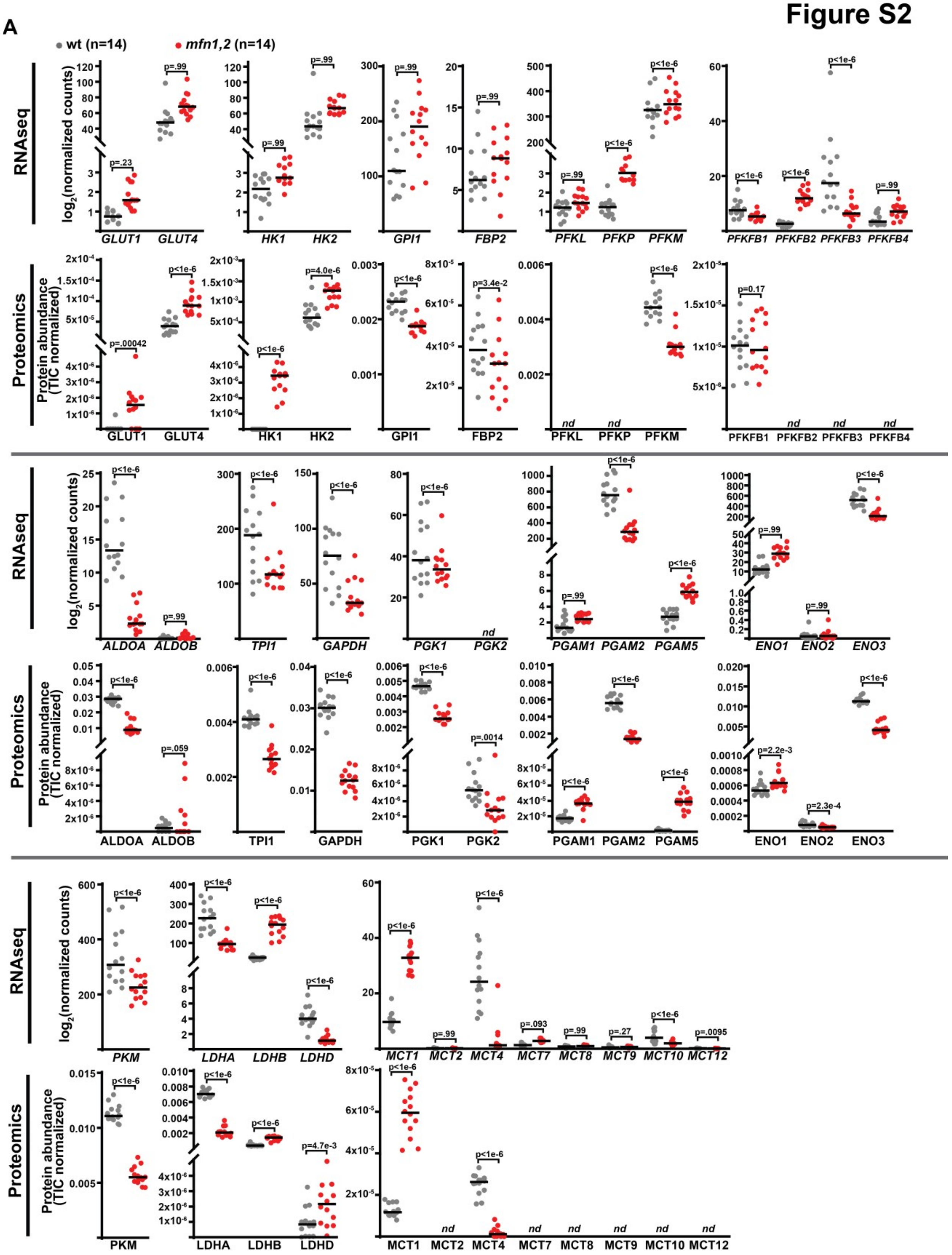
A
wt (n=14)
mfn1,2 (n=14)
RNAseq
log2(normalized counts)
GLUT1 GLUT4 HK1 HK2 GPI1 FBP2 PFKL PFKP PFKM PFKFB1 PFKFB2 PFKFB3 PFKFB4
p=.23 p=.99 p=.99 p=.99 p=.99 p=.99 p=.99 p<1e-6 p<1e-6 p<1e-6 p<1e-6 p<1e-6 p=.99
Proteomics
Protein abundance (TIC normalized)
GLUT1 GLUT4 HK1 HK2 GPI1 FBP2 PFKL PFKP PFKM PFKFB1 PFKFB2 PFKFB3 PFKFB4
p=.00042 p<1e-6 p<1e-6 p=4.0e-6 p<1e-6 p=3.4e-2 p<1e-6 p=0.17
nd nd nd nd nd
RNAseq
log2(normalized counts)
ALDOA ALDOB TPI1 GAPDH PGK1 PGK2 PGAM1 PGAM2 PGAM5 ENO1 ENO2 ENO3
p<1e-6 p=.99 p<1e-6 p<1e-6 p<1e-6 p=.99 p<1e-6 p<1e-6 p=.99 p=.99 p<1e-6
nd
Proteomics
Protein abundance (TIC normalized)
ALDOA ALDOB TPI1 GAPDH PGK1 PGK2 PGAM1 PGAM2 PGAM5 ENO1 ENO2 ENO3
p<1e-6 p=.059 p<1e-6 p<1e-6 p<1e-6 p=.0014 p<1e-6 p<1e-6 p<1e-6 p=2.2e-3 p=2.3e-4 p<1e-6
RNAseq
log2(normalized counts)
PKM LDHA LDHB LDHD MCT1 MCT2 MCT4 MCT7 MCT8 MCT9 MCT10 MCT12
p<1e-6 p<1e-6 p<1e-6 p<1e-6 p<1e-6 p=.99 p<1e-6 p=.093 p=.99 p=.27 p<1e-6 p=.0095
Proteomics
Protein abundance (TIC normalized)
PKM LDHA LDHB LDHD MCT1 MCT2 MCT4 MCT7 MCT8 MCT9 MCT10 MCT12
p<1e-6 p<1e-6 p<1e-6 p=4.7e-3 p<1e-6 p<1e-6
nd nd nd nd nd nd

Fig. S2. Levels of glycolytic enzymes in wild-type and *mfn1,2* TA muscle. (A) Log transformed transcript levels (Deseq2 normalized from RNAseq datasets) and Total Ion Count (TIC)-normalized protein abundances (from proteomics datasets) are presented for major muscle and alternative isoforms of glycolytic enzymes. P-values were calculated by multiple two-tailed t-tests (A) with adjustments for multiple comparisons.

Figure S3

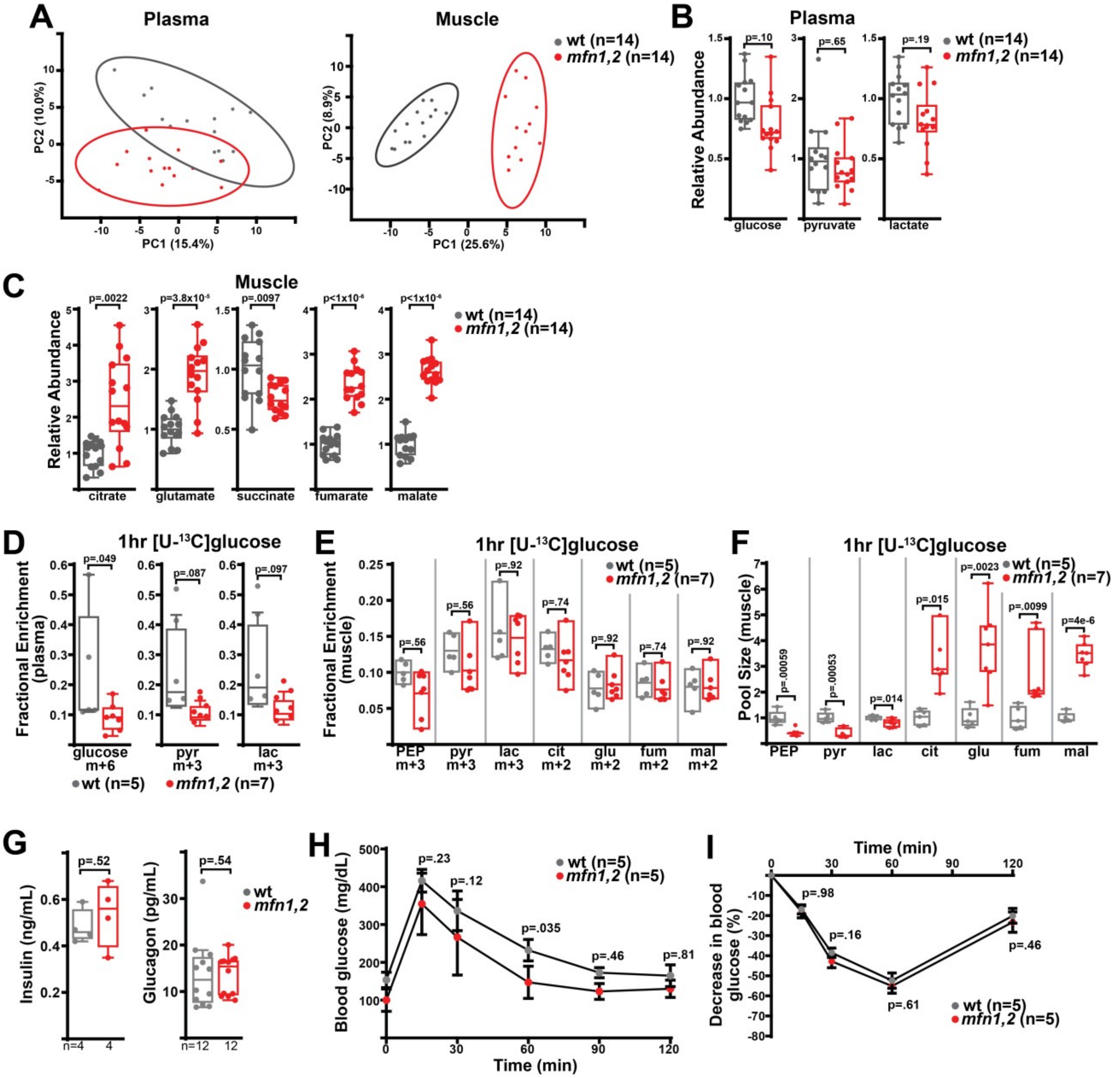

Fig. S3. Altered glucose utilization in *mfn1,2* muscle. (A) Principal component analysis of the plasma and TA muscle metabolomes from wt and *mfn1,2* animals. The percentage of total variance for principal components 1 and 2 (PC1 and PC2) are indicated on the x and y-axis. Ovals represent 95% confidence intervals. **(B)** Relative abundance of the indicated metabolites in plasma from wt and *mfn1,2* animals. **(C)** Relative abundance of the indicated TCA cycle metabolites in TA muscle from wt and

mfn1,2 animals. **(D)** Fractional enrichment of the indicated label species in plasma of the indicated genotype, following a 1 hr [U-^{13}C]glucose challenge. **(E)** Fractional enrichment of the indicated label species in muscle of the indicated genotype, following a 1 hr [U-^{13}C]glucose challenge. **(F)** Total pool sizes (normalized to wild-type levels) of the indicated label species in muscle of the indicated genotype, following a 1 hr [U-^{13}C]glucose challenge. **(G)** Plasma insulin and glucagon levels from fasted mice of the indicate genotypes. **(H)** Blood glucose following an oral glucose challenge (Glucose tolerance test) in fasted mice of the indicated genotypes. **(I)** Blood glucose following an insulin injection (Insulin tolerance test) in fasted mice of the indicated genotypes. In all panels, wild-type data are represented in gray, and *mfn1,2* data are represented in red. Box and whisker plots were plotted using the Tukey method. All data represent independent measurements from biological replicates. P-values were calculated by multiple two-tailed t-test (B-F), two-tailed t-test (G) or two-way ANOVA (H,I), with adjustments for multiple comparisons.

Figure S4

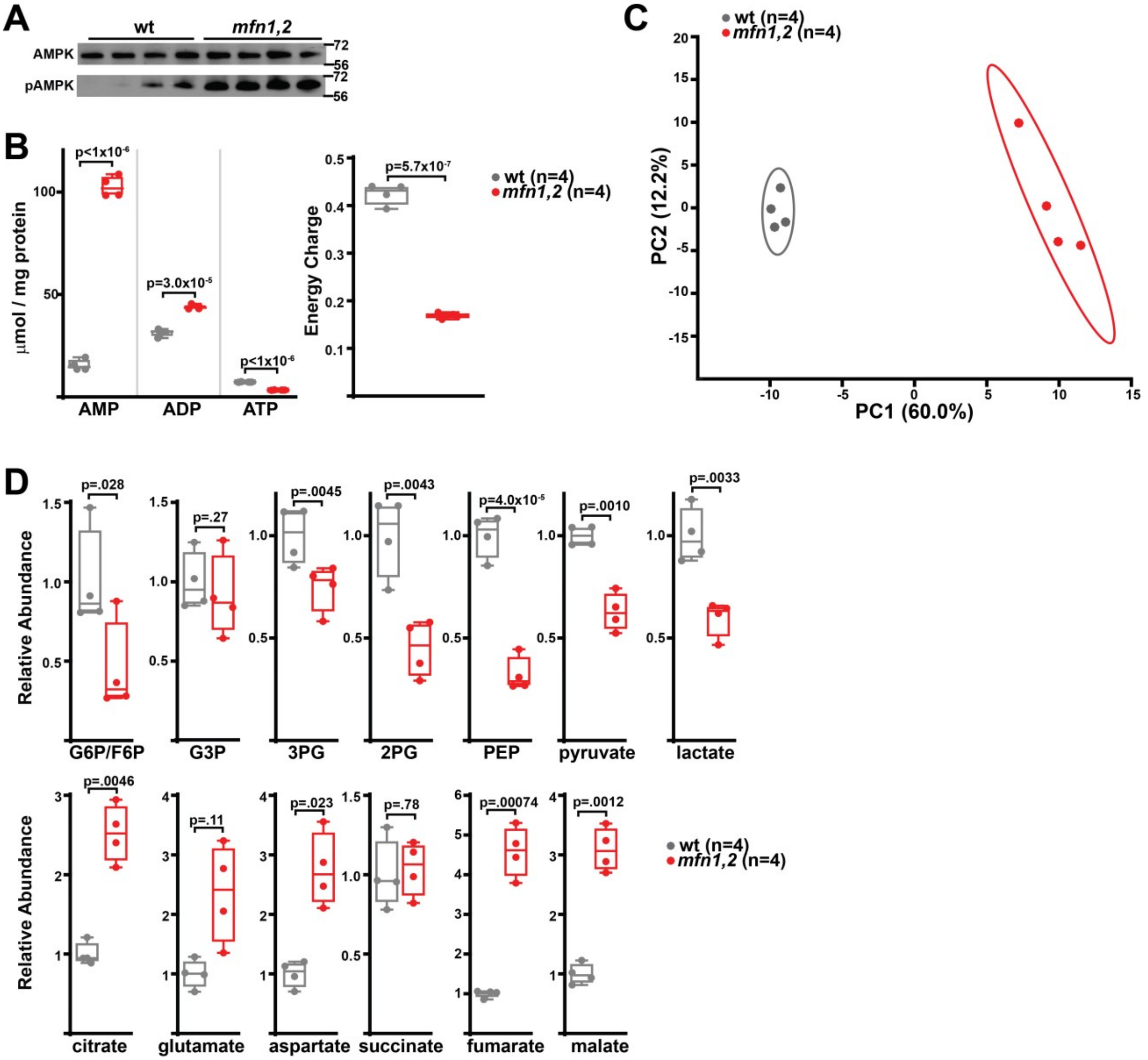

Fig. S4. Metabolic differences in exercised wild-type and *mfn1,2* mice. (A) Representative western blot for AMPK and phosphorylated AMPK (pAMPK) in indicated muscle samples from exercised mice. MW markers are indicated in kD. **(B)** On the left, absolute levels of AMP, ADP and ATP in wild-type and *mfn1,2* muscle of exercised mice, normalized to total protein amount. On the right, the energy charge ([ATP]+1/2[ADP])/([ATP]+[ADP]+[AMP]) in wild-type and *mfn1,2* muscle of exercised mice. **(C)** Principal component analysis of the TA muscle metabolome from wt and *mfn1,2*

exercised animals. The percentage of total variance for principal components 1 and 2 (PC1 and PC2) are indicated on the x and y-axis. Ovals represent 95% confidence intervals. **(D)** Relative abundance of the indicated glycolytic and TCA cycle metabolites in TA muscle from wt and *mfn1,2* exercised animals. In all panels, wild-type data are represented in gray, and *mfn1,2* data are represented in red. Box and whisker plots were plotted using the Tukey method. All data represent independent measurements from biological replicates. P-values were calculated by multiple two-tailed t-test (B,D) or two-tailed t-test (B), with adjustments for multiple comparisons.

Figure S5

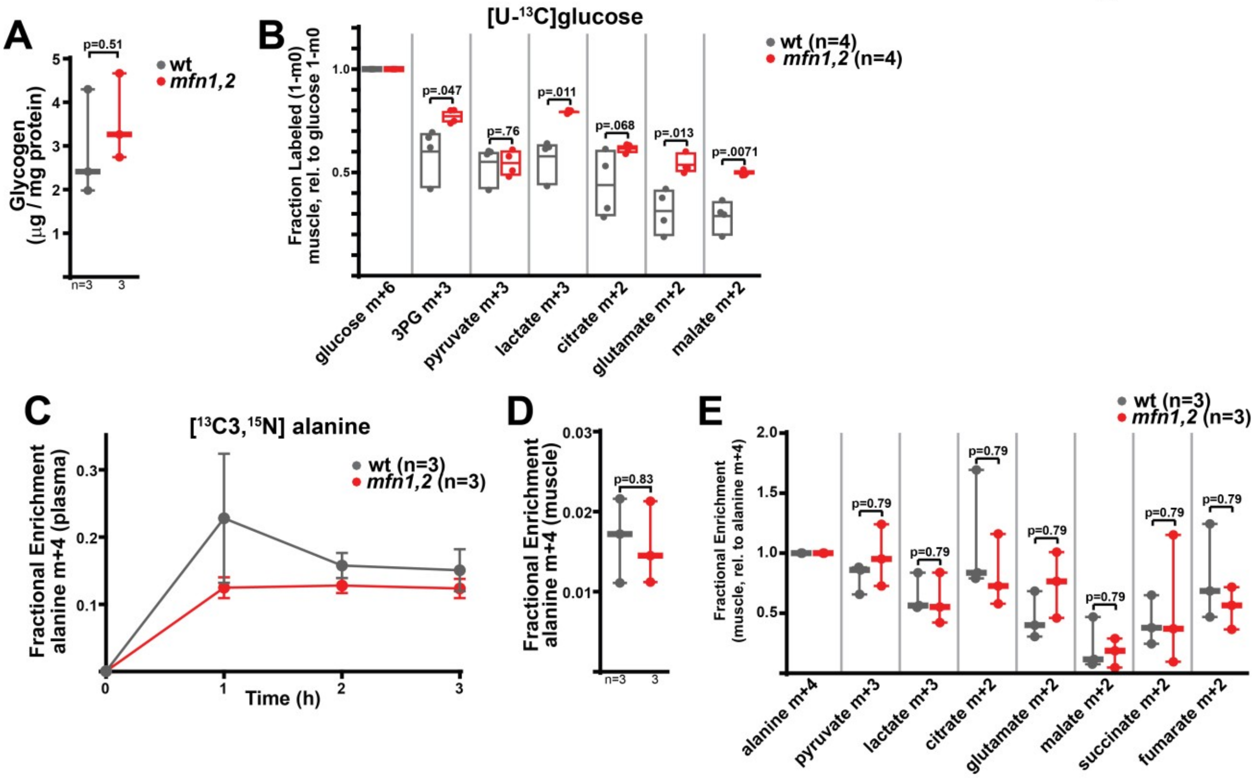

Fig. S5. Glucose and alanine utilization in wt and *mfn1,2* muscle. (A) Absolute levels of glycogen in wt and *mfn1,2* TA muscle, normalized to total protein content. **(B)** Total fractional enrichment (1-m0) of the indicated species in muscle from steady-state [U-^{13}C]glucose infusion. Data are normalized to total muscle glucose enrichment (1-m0) values. **(C)** Plasma labeling of alanine m+4 in steady-state infusion experiments with [^{13}C3,^{15}N]alanine. **(D)** Alanine m+4 enrichment in muscle from steady-state [^{13}C3,^{15}N]alanine infusions. Same color scheme as C. **(E)** Fractional enrichment of the indicated labeled species in muscle from steady-state [^{13}C3,^{15}N]alanine infusion. Data are normalized to muscle alanine m+4 enrichment values. In all panels, wild-type data are represented in gray, and *mfn1,2* data are represented in red. Box and whisker plots were plotted using the Tukey method. All data represent independent measurements from

biological replicates. P-values were calculated by multiple two-tailed t-test (B,E) or two-tailed t-test (A,D), with adjustments for multiple comparisons.

Figure S6

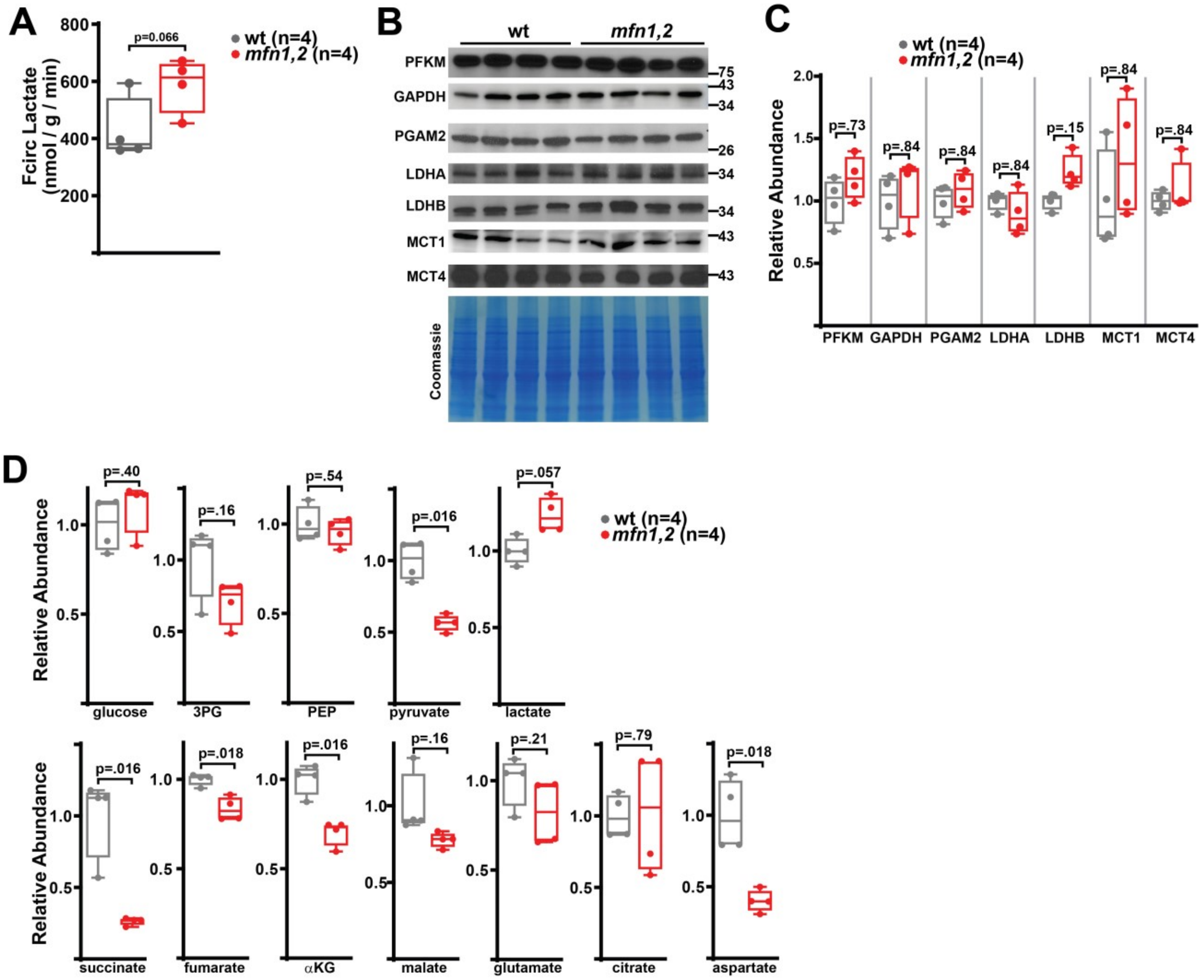

Fig. S6. Soleus muscle metabolism in wild-type and *mfn1,2* animals. (A) Circulatory turnover flux (Fcirc) for lactate in wild-type and *mfn1,2* animals, calculated as R*(1-L)/L where R indicates the infusion rate, and L indicates the steady-state plasma enrichment. **(B)** Abundances of the indicated proteins from wild-type and *mfn1,2* soleus muscle assessed by western blot. Coomassie stained proteins are shown as a loading control, and relative abundances are normalized to Coomassie staining. MW markers are indicated in kD. **(C)** Quantitation of protein abundance, based on western blot data in panel B. **(D)** Relative steady-state abundance of the indicated glycolytic and TCA cycle metabolites in wild-type and *mfn1,2* soleus muscle. In all panels, wild-type data are

represented in gray, and *mfn1,2* data are represented in red. Box and whisker plots were plotted using the Tukey method. All data represent independent measurements from biological replicates. P-values were calculated by multiple two-tailed t-test (C,D) or two-tailed t-test (A), with adjustments for multiple comparisons.

Figure S7

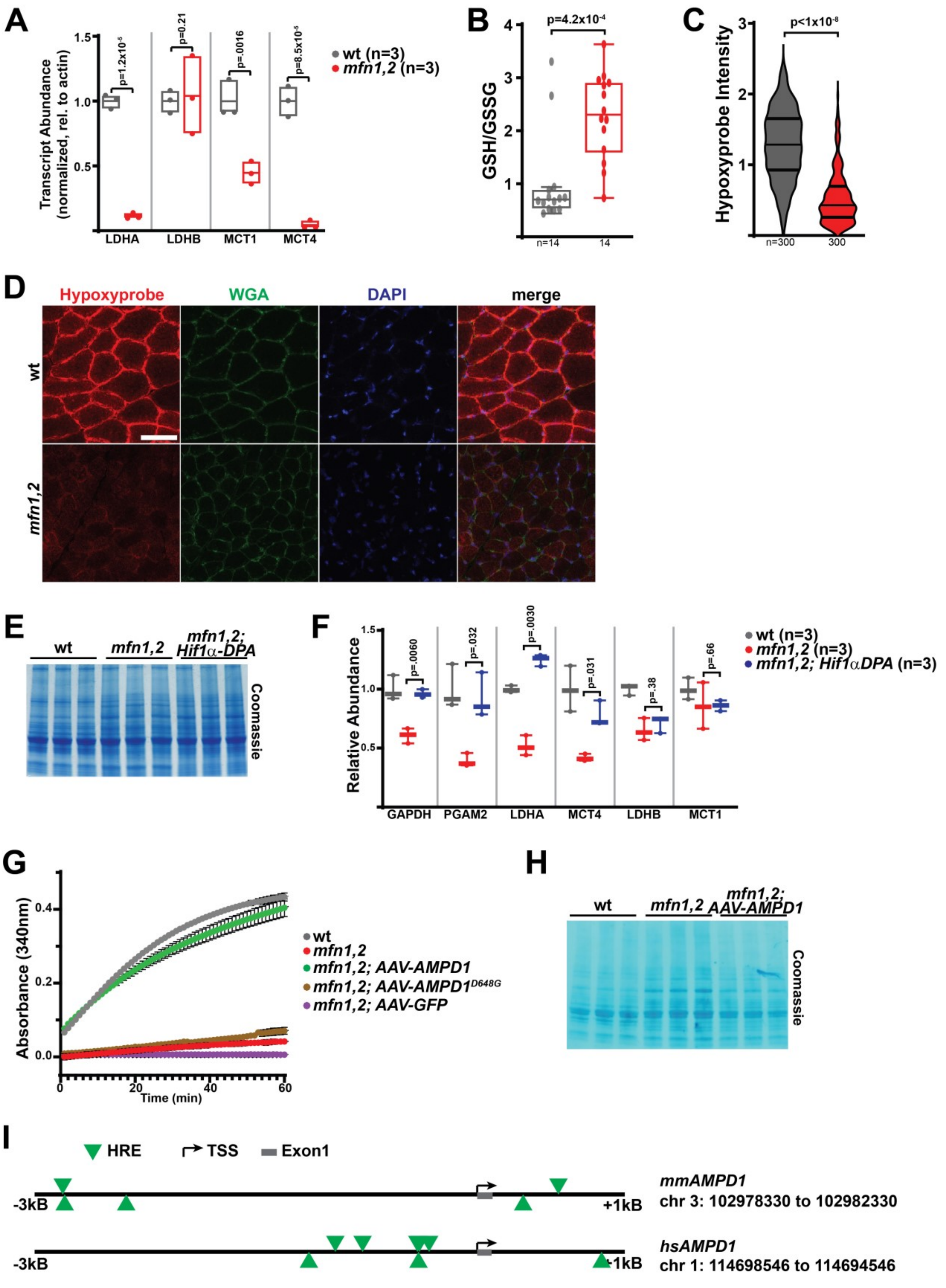
A
Transcript Abundance (normalized, rel. to actin)
LDHA
LDHB
MCT1
MCT4
wt (n=3)
mfn1,2 (n=3)
B
GSH/GSSG
p=4.2x10-4
n=14
14
C
Hypoxyprobe Intensity
n=300
300
D
Hypoxyprobe
WGA
DAPI
merge
wt
mfn1,2
E
wt
mfn1,2
mfn1,2; Hif1α-DPA
Coomassie
F
Relative Abundance
GAPDH
PGAM2
LDHA
MCT4
LDHB
MCT1
wt (n=3)
mfn1,2 (n=3)
mfn1,2; Hif1αDPA (n=3)
G
Absorbance (340nm)
Time (min)
wt
mfn1,2
mfn1,2; AAV-AMPD1
mfn1,2; AAV-AMPD1D648G
mfn1,2; AAV-GFP
H
wt
mfn1,2
mfn1,2; AAV-AMPD1
Coomassie
I
HRE
TSS
Exon1
-3kB
+1kB
mmAMPD1
chr 3: 102978330 to 102982330
hsAMPD1
chr 1: 114698546 to 114694546

Fig. S7. Hif1α and AMPD1 regulate metabolism in *mfn1,2* muscle. (A) Transcript abundance of the indicated genes from wt and *mfn1,2* TA muscle, as measured by qRT-PCR. **(B)** Relative ratios of GSH:GSSG in TA muscle tissue of the indicated genotypes. Same color scheme as A. **(C)** Hypoxyprobe intensity (based on reactivity of pimonidazole) was measured in 300 individual muscle fiber cross-sections from each of the indicated genotypes over three independent experiments. Same color scheme as A. **(D)** Representative cross-section images from wild-type and *mfn1,2* TA muscle, stained for pimonidazole adducts (Hypoxyprobe, red), muscle fiber boundaries (WGA, green) and nuclei (DAPI, blue). Scale bar; 50 μm. **(E)** Coomassie stained blot as a loading control for western blots in Fig.8C, 9B. **(F)** Quantitation of western blots from Fig. 7C; relative abundances are normalized to Coomassie staining. **(G)** Representative AMP deaminase activity assay from muscle lysates of the indicated genotype. Product formation is monitored by Absorbance at 340 nm over time. **(H)** Coomassie stained blot as a loading control for western blots in Fig. 9G. **(I)** Analysis of murine (mm) and human (hs) AMPD1 promoters. Sequences from -3kb to +1kb of the transcriptional start site (TSS) were searched for hypoxia response element (HRE) sequences: RCGTG, where R represents A or G. The positions of each identified HRE is indicated. Box and whisker plots were plotted using the Tukey method. All data represent independent measurements from biological replicates. P-values were calculated by multiple two-tailed t-test (A,B), Mann-Whitney (C), or one-way ANOVA (F), with adjustments for multiple comparisons.

Figure S8

A

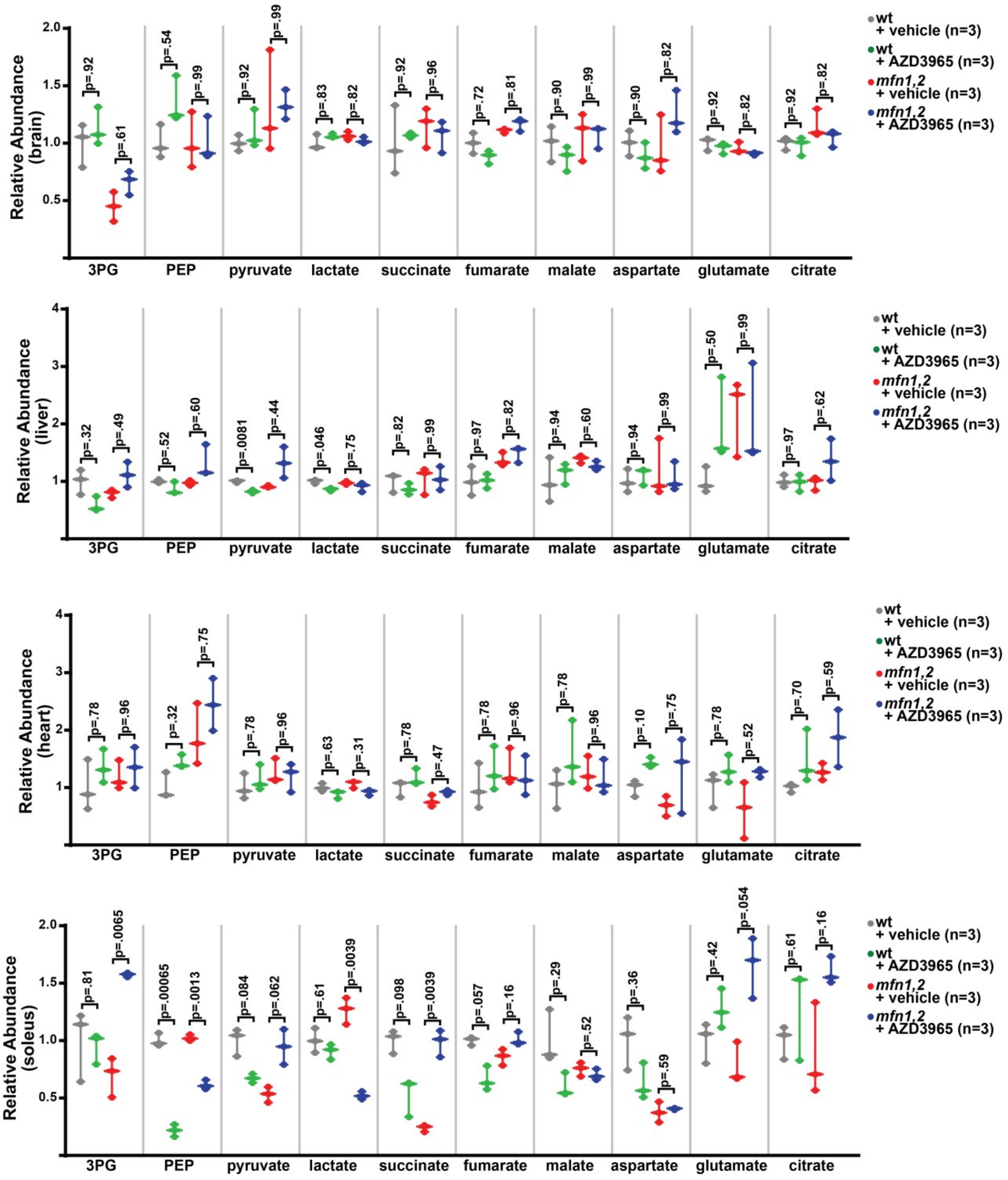

Fig. S8. Metabolic response to AZD3965 treatment in wild-type and *mfn1,2* animals.

(A) Relative steady-state abundance of the indicated metabolites in brain, liver, heart and soleus muscle of wild-type and mfn1,2 animals treated with vehicle of AZD3965 for three

days. Box and whisker plots were plotted using the Tukey method. All data represent independent measurements from biological replicates. P-values were calculated by multiple two-tailed t-test (A) with adjustments for multiple comparisons.

Data S1.

Table S1: RNAseq and Proteomics Analysis of *mfn1,2* and wild-type muscle.

Data S2.

Table S2: Plasma and muscle metabolomics analysis for *mfn1,2* and wild-type animals.

Data S3.

Table S3: Analysis of proteomic and transcriptomic data from case-control studies of patient cohorts with infantile mitochondrial myopathy (TK2-deficiency and RIRCD).
